# A Novel RAGE Modulator Induces Soluble RAGE to Reduce BACE1 Expression in Alzheimer's Disease

**DOI:** 10.1002/advs.202407812

**Published:** 2025-01-04

**Authors:** Seung‐Hyun Baek, Suji Hong, Eunae Kim, Sunyoung Park, Minyoung Lee, Jinsu Park, Yoonsuk Cho, Hyunjun Yoon, Daeseung Kim, Youngkwang Yun, Youbin Kim, Yoonjung Choi, Keunsoo Kang, Sangyong Jung, Jun Pyo Kim, Eunha Kim, Sang Won Seo, Yong‐Keun Jung, Dong‐Gyu Jo

**Affiliations:** ^1^ School of Pharmacy Sungkyunkwan University Suwon 16419 Republic of Korea; ^2^ Department of Molecular Science and Technology Ajou University Suwon 16499 Republic of Korea; ^3^ Deargen Inc. Daejeon 34051 Republic of Korea; ^4^ School of Biological Sciences Seoul National University Seoul 08826 Republic of Korea; ^5^ Department of Microbiology College of Science and Technology Dankook University Cheonan 31116 Republic of Korea; ^6^ Department of Medical Science College of Medicine CHA University Seongnam 13496 Republic of Korea; ^7^ Department of Neurology Samsung Medical Center Sungkyunkwan University School of Medicine Seoul 06355 Republic of Korea; ^8^ Alzheimer's Disease Convergence Research Center Samsung Medical Center Seoul 06351 Republic of Korea; ^9^ Neuroscience Center Samsung Medical Center Seoul 06351 Republic of Korea; ^10^ Biomedical Institute for Convergence at SKKU (BICS) Suwon 16419 Republic of Korea; ^11^ Department of Health Sciences and Technology SAIHST Sungkyunkwan University Seoul 06355 Republic of Korea; ^12^ Institute of Quantum Biophysics Sungkyunkwan University Suwon 16419 Republic of Korea

**Keywords:** 6‐Thioguanosine (6‐TG), Alzheimer's disease (AD), amyloid‐β (Aβ), BACE1, deep learning, drug repurposing, RAGE

## Abstract

β‐secretase (BACE1) is instrumental in amyloid‐β (Aβ) production, with overexpression noted in Alzheimer's disease (AD) neuropathology. The interaction of Aβ with the receptor for advanced glycation endproducts (RAGE) facilitates cerebral uptake of Aβ and exacerbates its neurotoxicity and neuroinflammation, further augmenting BACE1 expression. Given the limitations of previous BACE1 inhibition efforts, the study explores reducing BACE1 expression to mitigate AD pathology. The research reveals that the anticancer agent 6‐thioguanosine (6‐TG) markedly diminishes BACE1 expression without eliciting cytotoxicity while enhancing microglial phagocytic activity, and ameliorate cognitive impairments with reducing Aβ accumulation in AD mice. Leveraging advanced deep learning‐based tool for target identification, and corroborating with surface plasmon resonance assays, it is elucidated that 6‐TG directly interacts with RAGE, modulating BACE1 expression through the JAK2‐STAT1 pathway and elevating soluble RAGE (sRAGE) levels in the brain. The findings illuminate the therapeutic potential of 6‐TG in ameliorating AD manifestations and advocate for small molecule strategies to increase brain sRAGE levels, offering a strategic alternative to the challenges posed by the complexity of AD.

## Graphical abstract

This study demonstrates that 6‐thioguanosine (6‐TG) mitigates Alzheimer's disease (AD) pathology by binding directly to the Receptor for Advanced Glycation Endproducts (RAGE). This interaction downregulates BACE1 expression via the JAK2‐STAT1 pathway and increases soluble RAGE levels, thereby reducing amyloid‐beta accumulation and improving cognitive functions in AD animal models.

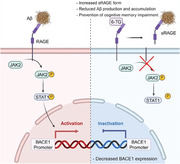

## 1 Introduction

Alzheimer's disease (AD), the predominant form of senile dementia, is characterized by a spectrum of neuropathological manifestations, including cognitive impairment, memory deficits, and comprehension dysfunction.^[^ ^1^ ^]^ The etiology of AD is multifactorial, with implicated factors ranging from Aβ accumulation,^[^ ^2^ ^]^ mitochondrial dysfunction,^[^ ^3^ ^]^ genetic factors (such as APOE^[^ ^4^ ^]^ and TREM2^[^ ^5^ ^]^), excessive oxidative stress,^[^ ^6^ ^]^ abnormal neuroimmune system responses,^[^ ^7^ ^]^ and the aggregation of neurofibrillary tangles.^[^ ^8^ ^]^ Among these factors, the amyloid cascade hypothesis has been a central focus of AD research for many years,^[^ ^9^ ^]^ proposing that Aβ accumulation is the initial trigger for neuronal dysfunction and cell death.^[^ ^10^ ^]^ In a healthy brain, the amyloid precursor protein (APP) undergoes sequential cleavage by α‐ and γ‐secretases, producing soluble APPα, P3, and AICD fragments.^[^ ^11^ ^]^ However, in the brains of individuals with AD, APP is truncated by β‐ and γ‐secretases, leading to the generation of soluble APPβ,^[^ ^9^ ^]^ AICD, and Aβ peptides ranging from 40 to 42 amino acids.

The critical factor distinguishing normal and abnormal APP processing is the enzyme BACE1. Mature BACE1 binds to and cleaves APP through its two catalytic aspartic sites.^[^ ^12^ ^]^ In the Alzheimer's brain, the expression and activity of BACE1 are reported to be 2–3 times higher than in the normal brain.^[^ ^13^ ^]^ BACE1 is highly sensitive to even slight stimulation due to its promoter, so it is prone to excessive upregulation, subsequently leading to increased Aβ production.^[^ ^14^ ^]^ However, despite the development of numerous BACE1 inhibitors aimed at disrupting its activity and reducing Aβ production, these drugs have faced challenges in clinical trials and failed to achieve commercialization.^[^ ^15^ ^]^ The primary reason for the failure of BACE1 inhibitors in clinical trials may be attributed to the essential functions BACE1 plays in a healthy brain.^[^ ^16^ ^]^ Several studies have suggested that BACE1 is involved in axon formation and neurodevelopmental processes,^[^ ^17^ ^]^ raising concerns about the potential side effects associated with complete BACE1 inhibition. Therefore, we propose a BACE1 regulation strategy that suppresses excessive BACE1‐mediated Aβ production while preserving its essential physiological functions.

As the RAGE engages with Aβ peptides across various neural interfaces including the blood–brain barrier (BBB), neuron, and microglia.^[^ ^18^ ^]^ Within the cerebrovascular endothelium, RAGE facilitates the translocation of circulating Aβ into the neural parenchyma,^[^ ^19^ ^]^ and in neurons, it promotes Aβ‐induced oxidative stress and disrupts intraneuronal transport, leading to mitochondrial dysfunction.^[^ ^18^, ^20^ ^]^ RAGE expression is significantly increased in an Aβ‐enriched environment, amplifying Aβ‐induced pathological responses at the BBB and in the brain.^[^ ^21^ ^]^ Furthermore, RAGE is known to mediate the upregulation of BACE1 expression in the context of neuroinflammatory responses.^[^ ^22^ ^]^ In addition to the strategies targeting the RAGE‐Aβ/AGEs interaction for AD, efforts have included using recombinant RAGE to decrease the activity of the RAGE pathway by acting as a decoy.^[^ ^18^, ^23^ ^]^ This approach aims to reduce neuroinflammation by capturing Aβ peptides, thereby preventing their interaction with cellular RAGE and mitigating the downstream effects that contribute to AD pathology. Given the limited success of directly inhibiting RAGE, promoting the conversion to soluble RAGE (sRAGE) may represent a novel and promising strategy in the development of AD therapeutics.^[^ ^23^, ^24^ ^]^

Considering the limitations of BACE1 inhibition, we propose a strategy modulating BACE1 expression to preserve its physiological functions while curtailing its pathological overactivity. In this study, we identified a drug 6‐TG that modulates BACE1 expression through its direct binding to the RAGE which activates via JAK2‐STAT1 pathway and increases sRAGE levels. This research not only contributes to the understanding of AD mechanisms but also presents a potential paradigm shift in therapeutic approaches, emphasizing gene regulation over enzyme inhibition.

## 2 Results

### 2.1 Inhibitory Effect of 6‐TG on the BACE1 Promoter Activity

**Figure 1:**
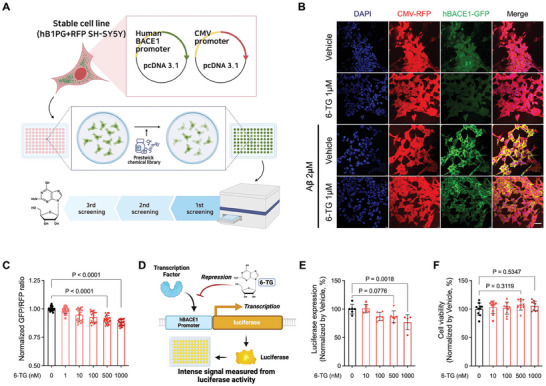
BACE1 promoter inhibition effect of 6‐TG. A) Overview of the drug screening system to identify a regulatory agent for the BACE1 promoter, such as 6‐TG. The methodology entails utilizing a stable cell line to express a BACE1‐GFP reporter construct, followed by the administration of a comprehensive drug library, and subsequent quantitative assessment of BACE1 promoter activity. B) Treatment with 6‐TG led to a reduction in GFP fluorescence, signaling a decrease in BACE1 activity, compared to the vehicle control. Red fluorescence delineates cellular morphology, while green fluorescence denotes BACE1 promoter driven GFP expression. Images were acquired using confocal microscopy at 40× magnification, with scale bars corresponding to 40 µm. C) Quantitative fluorescence analysis was conducted using a microplate reader to assess the effect of different concentrations of 6‐TG on BACE1 promoter activity. The ratio of green to red fluorescence (GFP/RFP) was calculated, providing a quantitative measure of the relative activity of the BACE1 promoter. D) Schematic representation of the luciferase reporter assay, employed for assessing transcriptional activity. E) Dose‐dependent repression of BACE1 transcription by 6‐TG in HEK293T cells is depicted, with luciferase expression normalized to Renilla luciferase activity serving as an internal control. F) Cell viability was evaluated using MTT assay, confirmed the nontoxicity of 6‐TG across various concentrations. Data are presented as mean (SD) with *P* values derived from ordinary one‐way ANOVA and Holm–Šídák's multiple comparisons test, denoting statistical significance relative to vehicle‐treated controls.

To identify agents that can modulate BACE1 promoter activity, we developed a fluorescence‐based reporter system. This system serves as a reliable surrogate for the human BACE1 promoter's activity. By integrating the human BACE1 promoter sequence (Figure S1A, Supporting Information) into the pDNA3.1‐EGFP vector, we constructed a plasmid (phB1PG, Figure S1B,C, Supporting Information) that facilitates the expression of green fluorescent protein (GFP) as a direct readout of BACE1 promoter activation. Subsequently, we utilized the Prestwick Chemical Library, which encompasses FDA‐approved drugs, to screen for potential modulators using the hB1PG‐RFP SH‐SY5Y cell line. Through three independent experimental runs, we discerned five pharmacological agents that exhibited a discernible decrease in GFP output, indicative of suppressed BACE1 promoter activity. Notably, (−)‐2‐amino‐6‐mercaptopurine riboside hydrate (6‐TG), a metabolite generated during the metabolism of thioguanine,^[^ ^25^ ^]^ was among these candidates (**Figure** **1**A).

To elucidate the regulatory effects of 6‐TG on BACE1 expression, we treated varying concentrations of 6‐TG to the hB1PG‐RFP SH‐SY5Y cell line. Quantitative analyses demonstrated significant suppression of GFP expression in cells treated with 6‐TG compared to the vehicle group, signifying a downregulation of BACE1 expression (Figure 1B,C; and Figure S1D, Supporting Information). BACE1 promoter assay substantiated that the inhibition of promoter activity was attributable to 6‐TG, with a significant reduction observed at dosages exceeding 100 nm (Figure 1D,E; and Figure S1E, Supporting Information). Although 6‐TG is utilized in leukemia therapy and is recognized for its proapoptotic properties,^[^ ^26^ ^]^ our cell viability assays revealed no significant cytotoxicity at therapeutically effective concentrations (Figure 1F).

### 2.2 Comparison of the BACE1 Reducing Effect of 6‐TG and Its Analogues

**Figure 2:**
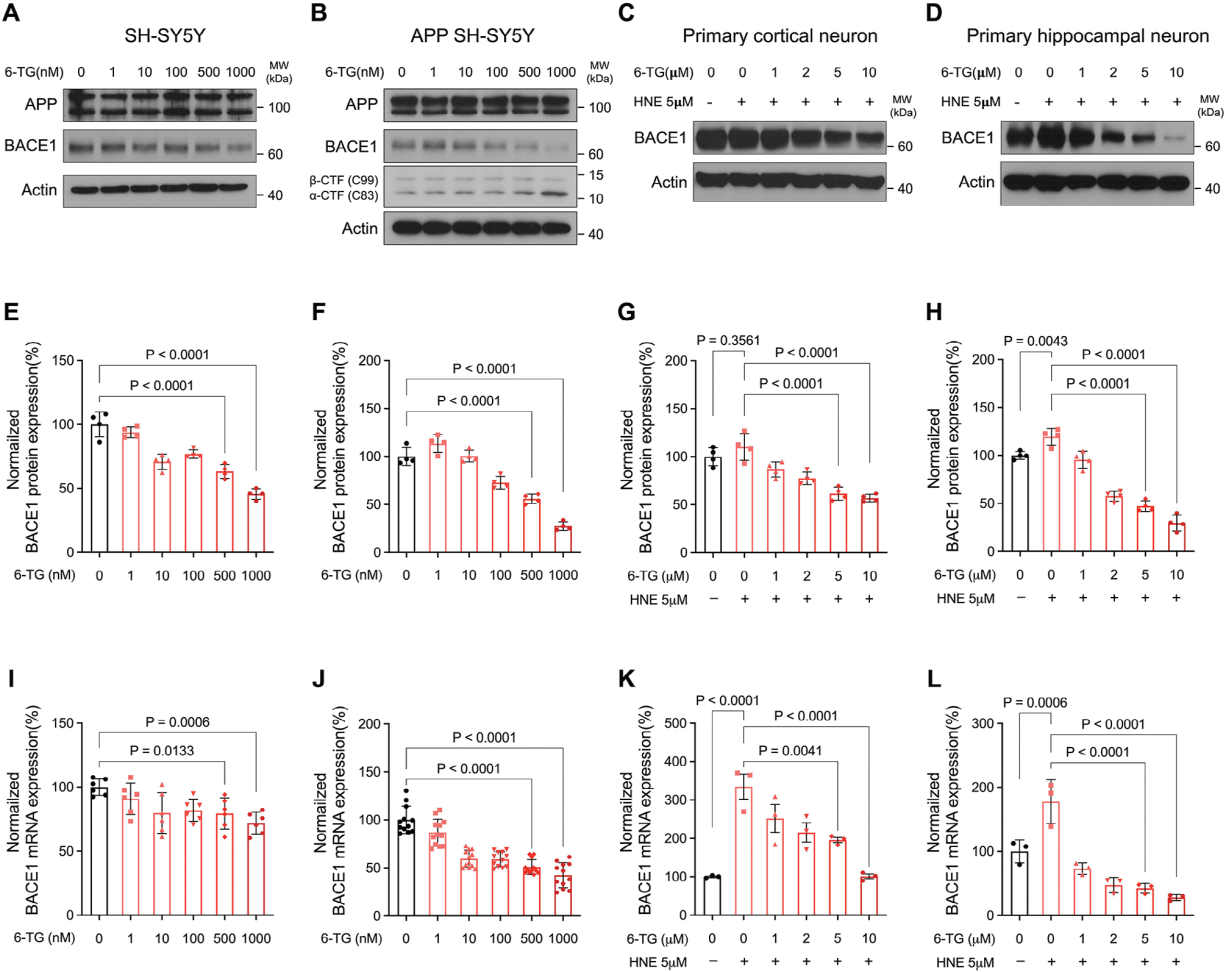
Modulation of BACE1 expression by 6‐TG in neuronal cell. A) SH‐SY5Y cells treated with varying concentrations of 6‐TG for 24 h show a dose‐dependent reduction in BACE1 protein levels. B) APP‐SH‐SY5Y cells were treated with 6‐TG or vehicle control, with detection of APP, BACE1, and APP cleavage products C99 and C83. C,D) Rat primary neurons (cortical and hippocampal) were treated with 5 µm HNE to induce BACE1 expression, followed by 24 h exposure to 6‐TG or vehicle control. E–H) Quantification of BACE1 expression showing correspondence from A–E, B–F, C–G, D–H. I,J) Quantitative PCR determination of BACE1 mRNA levels in SH‐SY5Y and APP‐SH‐SY5Y cells post 6‐TG treatment with analysis using three different primer sets. K,L) Consolidated data from two primer sets across three independent experiments, demonstrating the reproducibility and significance of the results. Data are presented as mean (SD). Statistical significance is indicated for comparisons against vehicle‐treated E,F,I,J) and HNE‐treated G,H,K,L) controls, using ordinary one‐way ANOVA and Holm–Šídák's multiple comparisons test.

To confirm the 6‐TG‐induced decrease in BACE1 expression, we performed a series of dose‐response experiments in SH‐SY5Y cells. These results showed that 6‐TG attenuated BACE1 protein and mRNA levels in a concentration‐dependent manner (**Figure** **2**A,E,I). 6‐TG is one of the purine analogues and there are many substances showing structural similarity.^[^ ^25^ ^]^ Therefore, we next investigated whether other purine analogues could also regulate BACE1 expression like 6‐TG. To identify the molecular determinants of 6‐TG that modulate BACE1 expression, we performed a comparative analysis between 6‐TG and its structural analogues (Figure S2A,F, Supporting Information). We observed that 6‐TG was more effective than its analogues in reducing BACE1 expression, which was induced by the lipid peroxidation product, 4‐hydroxy‐2‐nonenal (HNE),^[^ ^13^ ^]^ or Aβ. Notably, compounds within the structurally similar analogue group also demonstrated partial reductions in the expression of BACE1 (Figure S2B,C,E,F, Supporting Information). This finding suggests a link between the structural characteristics of 6‐TG and its ability to regulate the BACE1 promoter.

We investigated BACE1 expression in neuronal cells overexpressing amyloid precursor protein (APP). A stepwise reduction in BACE1 expression was observed with increasing doses of 6‐TG (Figures 2B,F). This trend was consistent with a corresponding decrease in mRNA levels (Figure 2J). Notably, a dose of 1 µm 6‐TG appeared to normalize the aberrant amyloidogenic processing of APP, as indicated by increased CTF‐α levels (Figure 2B). To determine whether the increase in CTF‐α levels induced by 6‐TG was due to a reduction in BACE1 expression or increased α‐secretase activity, we conducted further studies using BACE1 and γ‐secretase inhibitors. Under these conditions, no significant changes in CTF‐α levels were observed in response to 6‐TG treatment (Figure S2G, Supporting Information). It is well established that a reduction in BACE1 activity shifts APP processing toward the α‐secretase pathway, leading to increased CTF‐α levels.^[^ ^27^ ^]^ Consistent with this, our results showed that CTF‐α levels increased upon treatment with the BACE1 inhibitor in the absence of 6‐TG. Therefore, these results suggest that 6‐TG does not exert an agonistic effect on α‐secretase but rather increases CTF‐α levels through the downregulation of BACE1 expression. Taken together, 6‐TG reduces BACE1 expression and counteracts the pathological hallmarks of AD.

To verify that the suppression of BACE1 by 6‐TG was independent of its effects on cell death, we treated primary neuronal cultures (from both the cortex and hippocampus) with 6‐TG following exposure to HNE. Remarkably, administering 6‐TG in a dose‐dependent manner led to a significant decrease in BACE1 expression both at the protein (Figure 2C,D,G,H) and mRNA levels (Figure 2K,L). Furthermore, electrophysiological assessments of primary neurons treated with 1 µm 6‐TG, using patch‐clamp techniques, showed no significant differences compared to neurons treated with vehicles (Figure S3A,B, Supporting Information). This suggests that the effect of 6‐TG on BACE1 expression does not alter the fundamental intrinsic electrophysiological properties of neurons. Collectively, these results support the notion that 6‐TG specifically regulates BACE1 expression in neuronal cells, highlighting its potential as a targeted modulator of BACE1.

### 2.3 6‐TG Ameliorates Cognitive Deficits in APP/PS1 Mice

**Figure 3:**
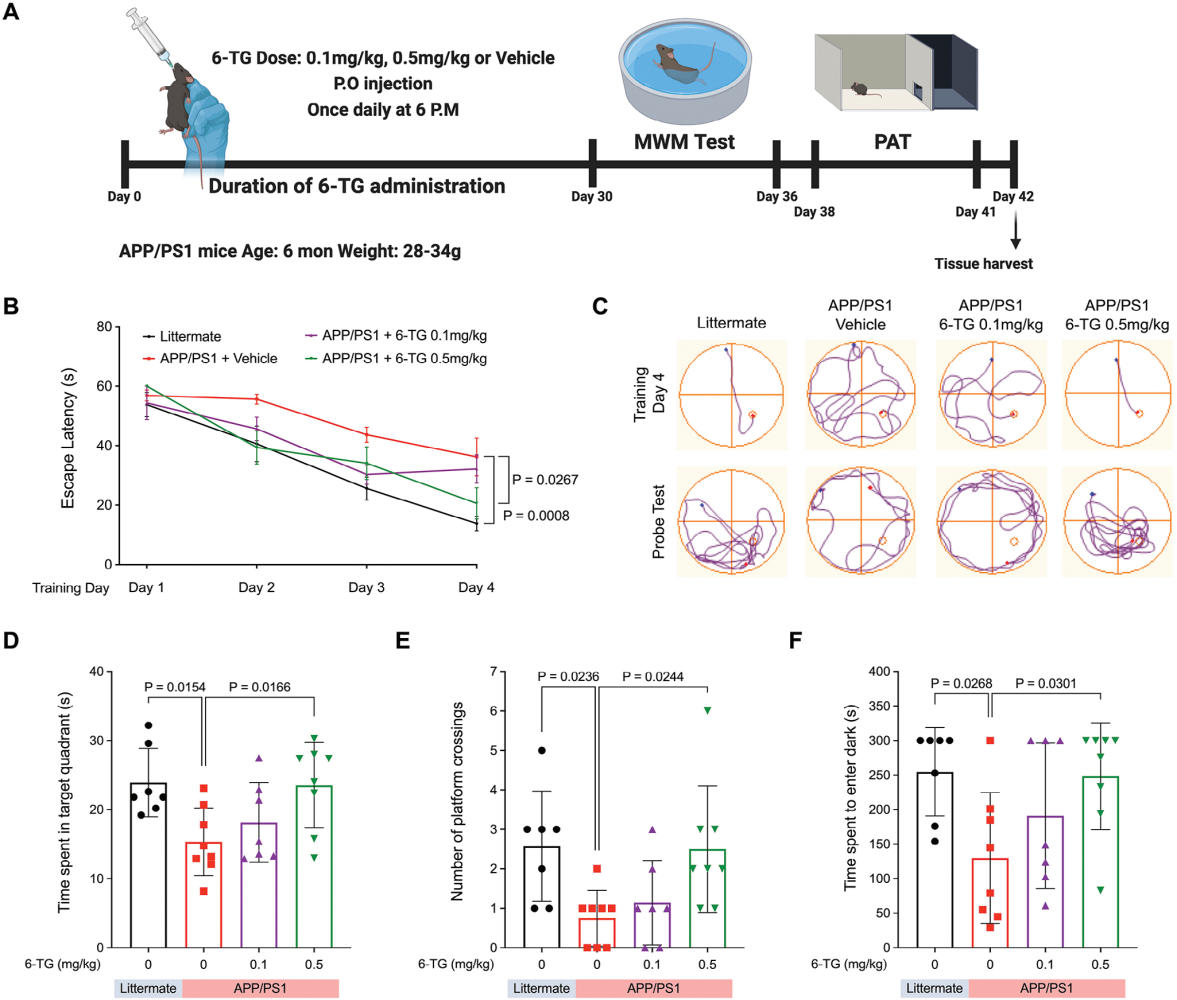
6‐TG mitigates cognitive impairments in APP/PS1 AD mouse model. A) Experimental timeline and treatment regimen. Six‐month‐old APP/PS1 mice and wild‐type littermates were administered orally with 6‐TG (0.1, 0.5 mg kg^−1^) or vehicle (DW: 1994 DMSO 500:1) daily for 30 days. Cognitive functions were evaluated using Morris water maze (MWM) to assess spatial learning, with escape latency ensured over four trials daily. Two days post‐MWM, fear‐associated memory was assessed using the passive avoidance test (PAT). (Sample size: WT + Vehicle *n* = 7, APP/PS1 + Vehicle *n* = 8, APP/PS1 + 6‐TG 0.1 mg kg^−1^ *n* = 7, APP/PS1 + 0.5 mg kg^−1^ *n* = 8). B) Escape latency trends across MWM trials. C) Representative swim paths from day 4 of learning or probe trials, depicting 1‐min navigational patterns. D) Analysis of time spent in the target quadrant, reflective of long‐term memory retention. E) Platform crossings incidents, compared across treatment groups. F) Latency to dark chamber entry in PAT, indicative of fear‐associated memory retention. Data are presented as mean (SD). Statistical analyses performed with ordinary one‐way ANOVA and Holm–Šídák's multiple comparisons test, with *P* values denoting significance against APP/PS1 vehicle‐treated controls.

To explore the potential of 6‐TG in improving cognitive function, we conducted an in vivo evaluation using APP/PS1 mice. The mice were orally given 6‐TG at a nontoxic dose (Figure S4A, Supporting Information), followed by a series of behavioral tests to assess their learning and memory capabilities. One key test was the Morris water maze (MWM), a well‐established tool for evaluating spatial learning and memory (**Figure** **3**A). Notably, during the MWM test, the APP/PS1 mice treated with 0.5 mg kg^−1^ 6‐TG showed cognitive performance comparable to that of their healthy littermates over a 4‐day training period. This indicates a significant improvement in learning deficits usually observed in vehicle‐treated APP/PS1 mice (Figure 3B). Further evidence of cognitive enhancement was observed in the probe test, where 6‐TG‐treated APP/PS1 mice displayed quicker and more accurate navigation toward the previously hidden platform compared to the vehicle‐treated APP/PS1 mice (Figure 3C). In the probe trial, both the littermate controls and the 6‐TG‐treated APP/PS1 mice spent significantly more time in the target quadrant, suggesting better spatial memory retention that the vehicle‐treated APP/PS1 group (Figure 3D). Additionally, the 6‐TG‐treated APP/PS1 mice had an increased number of platform crossings, reinforcing the positive impact of 6‐TG on spatial memory (Figure 3E).

The passive avoidance test was also used to assess memory retention further. Mice treated with 6‐TG showed significantly longer latencies before entering a dark chamber, indicating enhanced fear conditioning memory (Figure 3F). These in vivo results not only demonstrate the neuroprotective properties of 6‐TG but also support its potential as a therapeutic option for AD.

### 2.4 6‐TG Attenuates BACE1 Expression and Aβ Accumulation in APP/PS1 Mice

**Figure 4:**
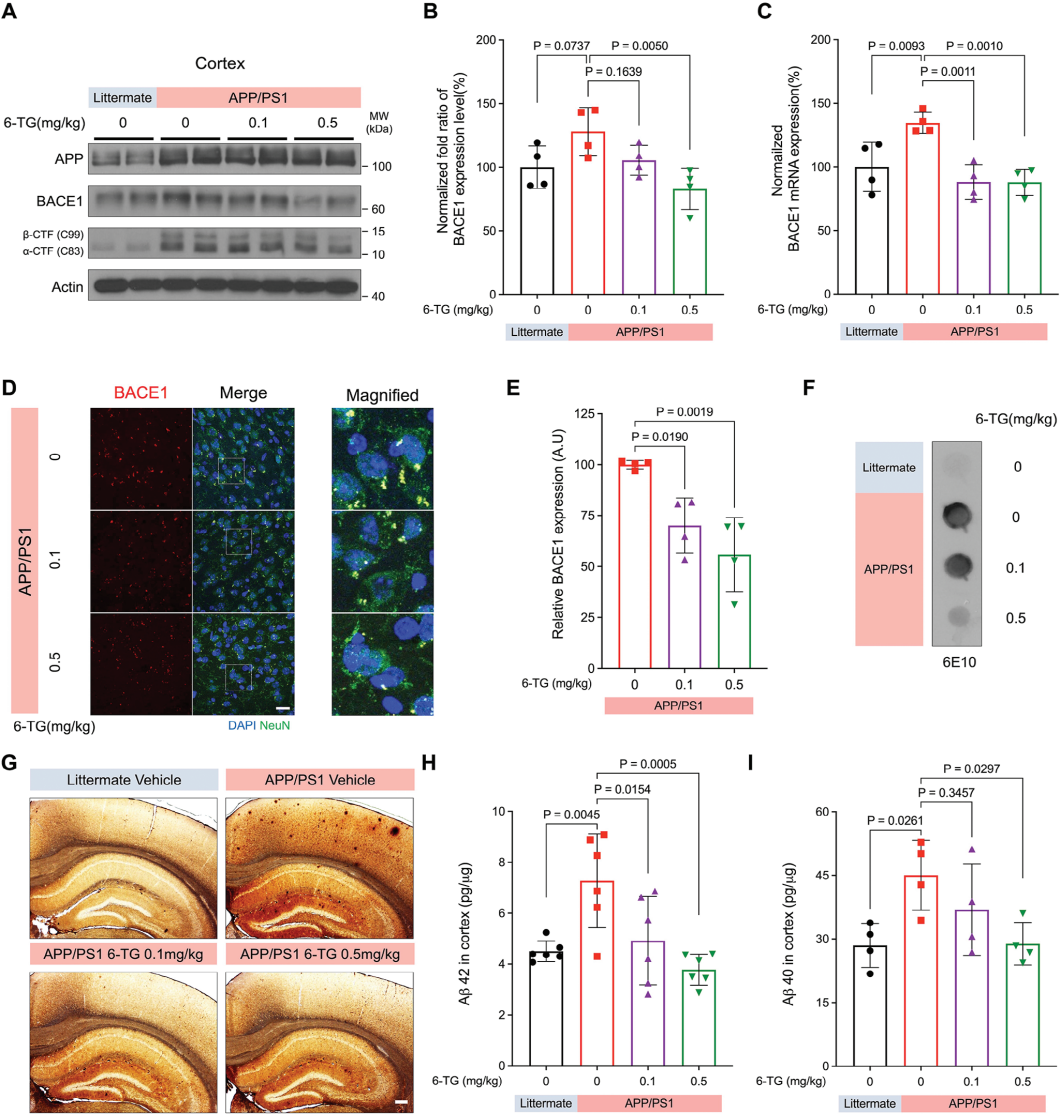
6‐TG attenuates AD pathology in APP/PS1 mice. A) Representatives immunoblots of BACE1 and APP C‐terminal fragments, cortical tissues collected after behavioral assessments, lysed in T‐PER tissue buffer, with proteins in the supernatant analyzed. B) Quantification of BACE1 protein levels, normalized to β‐actin. C) BACE1 mRNA levels in cortical tissues assessed by quantitative PCR, normalized to GAPDH expression, using three primer sets. D) Representative immunofluorescence of cortical BACE1 (red) and neuronal marker NeuN (green) expression, captured under a 40× objective. Scale bar = 20 µm. E) Quantification analysis of BACE1 expression from immunofluorescent imaging D). F) Dot blot analysis for Aβ detection using 6E10 antibody on 10 µg of cortical protein loaded on nitrocellulose membrane. G) DAB staining of Aβ plaques in 30 µm. Brain sections using an Abcam kit with 6E10 as the primary antibody and blind evaluations of hippocampal and cortical regions under a 4× objective. Scale bar = 80 µm. H,I) ELISA quantification of Aβ level in soluble brain fractions, employing standard curve comparison. Data are presented as mean (SD). Statistical analysis was conducted using ordinary one‐way ANOVA and Holm–Šídák's multiple comparisons test, with significance assessed against APP/PS1 vehicle‐treated groups.

After showing that 6‐TG improves cognition, we examined its effects on the brain pathology of APP/PS1 mice. We analyzed BACE1 protein levels in the cortex and hippocampus, key areas affected by AD. APP/PS1 mice treated with 0.5 mg kg^−1^ of 6‐TG showed a significant decrease in BACE1 expression (**Figure** **4**A,B; and Figure S4B,C, Supporting Information) and its product C99 (Figure 4A), indicating reduced activity of the enzyme responsible for cutting APP into Aβ. This reduction was confirmed at the RNA level (Figure 4C; and Figure S4D, Supporting Information) and through immunofluorescence assays that showed less BACE1 throughout the brain (Figure 4D,E; and Figure S4E,F, Supporting Information).

Given the role of Aβ in AD, we hypothesized that reducing BACE1 with 6‐TG would lead to fewer Aβ deposits. Indeed, dot blot analyses showed a significant reduction in Aβ, most notably in the cohort administered with 0.5 mg kg^−1^ 6‐TG (Figure 4F; and Figure S4G, Supporting Information). Furthermore, APP/PS1 mice treated with 6‐TG showed a marked decrease in Aβ plaque deposition compared to vehicle‐treated APP/PS1 mice (Figure 4G). Quantitative ELISA assays further confirmed substantive declines in both Aβ_42_ and Aβ_40_ peptides in the 6‐TG‐treated APP/PS1 mice (Figure 4H,I; and Figure S4H,I, Supporting Information). This comprehensive decrease in both Aβ plaques and soluble Aβ forms supports the idea that 6‐TG has a broad neuroprotective effect (Figure S4J, Supporting Information), reducing key Alzheimer's markers and offering a potential treatment strategy.

### 2.5 Deciphering 6‐TG Neuroprotective Mechanisms in AD through Transcriptomic Analysis and Target Prediction

**Figure 5:**
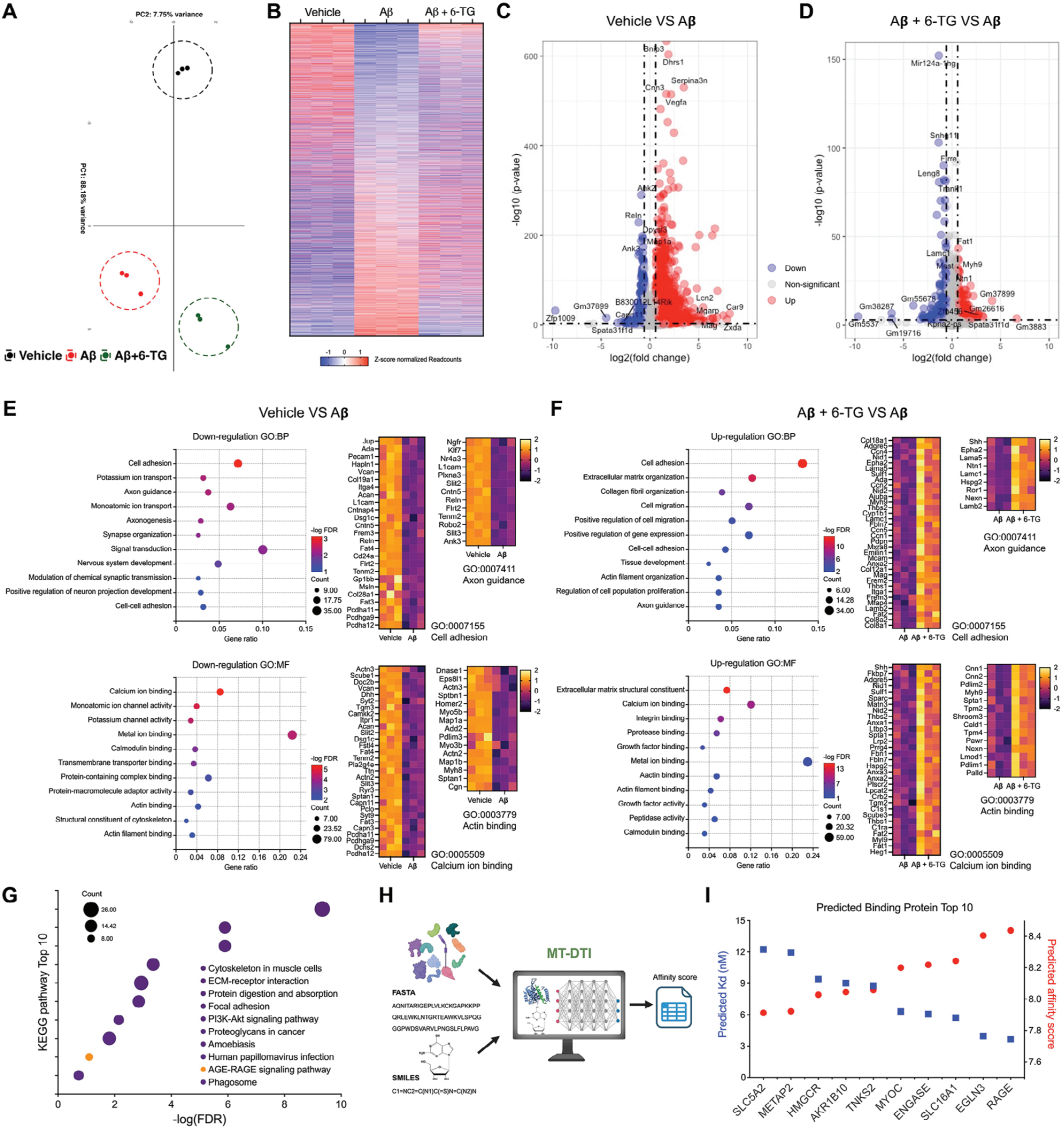
Identifying RAGE as a potential target for 6‐TG using the MT‐DTI model. A) Principal component analysis (PCA) displaying the multidimensional distribution of samples based on differentially expressed genes (DEGs) in mouse primary neurons. Points represent individual samples: vehicle‐treated (black), Aβ‐treated (red), and Aβ plus 6‐TG‐treated (green), with principal components 1 and 2 explaining 88.18% and 7.75% of the variation, respectively, delineating the distinct groupings. B) Heatmap showcasing hierarchical clustering of DEGs among treatments. Expression level variations are color‐coded: red for increased, blue for decreased, normalized to z‐score read counts, illustrating shifts relative to Aβ treatment. C,D) Volcano plots depicting DEGs between Vehicle versus Aβ and Aβ versus Aβ + 6‐TG groups, with significantly upregulated genes highlighted in red and downregulated genes in blue based on log2(fold change) and −log10(*p*‐value). E,F) Gene Ontology (GO) analysis showing significantly altered biological processes and molecular functions affected by Aβ E) or 6‐TG treatment F), represented by gene ratios and enrichment significance (−log FDR value). G) Kyoto Encyclopedia of Genes and Genomes (KEGG) pathway analysis highlighting the key pathways modulated by 6‐TG in Aβ‐treated neurons, quantified by FDR values and gene counts. H) Schematic representation of the MT‐DTI deep learning model used for predicting potential interactions between 6‐TG and target proteins. I) MT‐DTI model‐derived predictive binding affinities for top ten targets, dual *y*‐axis plot indicating predicted dissociation constant (*K*_d_) and predicted affinity score, denoting binding strength. Blue squares represent predicted *K*_d_ and red circles indicate predicted affinity score, across identified protein targets. E–G) Analyzes in detail the gene expression variations following 6‐TG treatment in Aβ‐challenged neurons, with a focus on fold changes greater than 1.5 and a false discovery rate (FDR) less than 0.05.

To elucidate the molecular mechanisms underlying the neuroprotective effects of 6‐TG in the context of Aβ induced neurotoxicity, we conducted RNA sequencing (RNA‐seq) analyses on primary mouse neurons (**Figure** **5**A–G; and Figure S5A,B, Supporting Information) and human embryonic stem cell (hESC)‐derived neurons (Figure S5C–G, Supporting Information). This transcriptomic assessment revealed significant changes in gene expression patterns upon exposure to Aβ and subsequent treatment with 6‐TG (Figure 5A,B,D). Among the 14511 genes examined, a substantial alteration in the expression profile was observed, with 293 genes upregulated and 313 genes downregulated in response to 6‐TG treatment in Aβ‐challenged neurons, demonstrating a comprehensive genomic response (Fold Change >1.5, *p*‐value <0.05, Figure 5D). Gene ontology (GO) enrichment analysis of these differentially expressed genes (DEGs) highlighted significant changes primarily in the domains of extracellular matrix (ECM) organization and protein binding activities (Figure 5F; and Figure S5B, Supporting Information).

To conduct an in‐depth analysis of the cellular effects of 6‐TG and establish its underlying mechanisms, we particularly focused on DEGs that were downregulated in the Aβ treatment group but upregulated in the 6‐TG + Aβ treatment group. By performing a comparative analysis between these groups, we aimed to identify specific pathways and processes influenced by 6‐TG. GO analysis of these DEGs revealed that 6‐TG treatment reversed the expression patterns of GO terms related to Axon Guidance (Biological Process) and Actin Binding (Molecular Function). These comparative analysis estimate that 6‐TG may alleviate Aβ‐induced toxicity, thereby preserving cytoskeletal integrity and maintaining cellular structure. Moreover, Kyoto Encyclopedia of Genes and Genomes (KEGG) pathway analysis of DEGs revealed a pronounced impact on pathways associated with the extracellular response (ECM‐receptor interaction), with a particular emphasis on the signaling pathway mediated by RAGE, marking it as a significantly modulated pathway (Figure 5G; and Figure S5H,I, Supporting Information).

To further corroborate these findings and identify potential molecular targets of 6‐TG, we employed a deep learning‐based tool, Molecule Transformer Drug Target Interaction Model (MT‐DTI),^[^ ^28^ ^]^ which predicts drug‐target interactions based on the chemical structures (Simplified molecular‐input line‐entry system, SMILES) and amino acid sequences (FASTA) of a target protein (Figure 5H). This predictive model pinpointed several proteins as potential high‐affinity targets for 6‐TG, notably identifying RAGE as the most significant target, supported by strong binding predictions and an estimated dissociation constant (*K*_d_ = 3.657 nm, Figure 5I). These transcriptomic insights not only illuminate the intricate genomic response modulated by 6‐TG but also suggest a key target interaction, providing a valuable framework for understanding the therapeutic potential of 6‐TG in mitigating the pathological features of AD.

### 2.6 6‐TG Modulates BACE1 Expression via RAGE Pathway in AD Models

**Figure 6:**
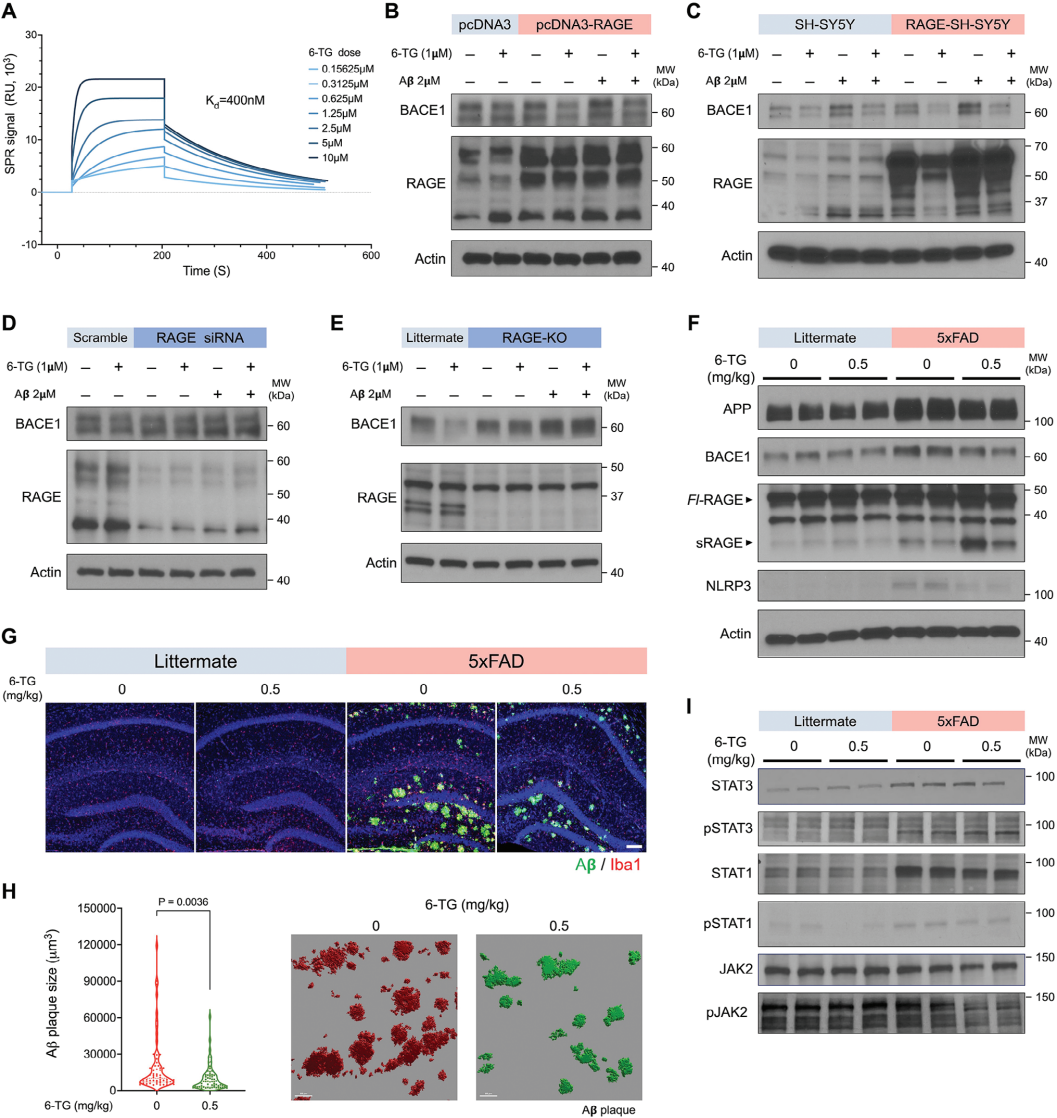
Effects of 6‐TG on the RAGE‐BACE1 axis in AD models. A) Surface plasmon resonance (SPR) analysis illustrating the interaction kinetics between immobilized RAGE protein and 6‐TG at varying concentrations (0.15 625 to 10 µm). The sensorgram facilitated deprivation of association/dissociation rate constants were derived from the sensorgram, and the equilibrium dissociation rate constants and the equilibrium dissociation constant (*K*_d_), highlighting a 1:1 binding model between response units (RU) and 6‐TG concentration. B,D) Alteration in BACE1 protein expression investigated in SH‐SY5Y cells modulating RAGE expression: overexpression B) and suppression D), followed by 6‐TG and Aβ treatment to observe BACE1 level changes. C) Comparative analysis of BACE1 protein levels in neuronal cells under RAGE overexpression, assessing the modulation of BACE1 by 6‐TG and Aβ. E) Protein expression studies in neuronal cells from RAGE knockout mice, with A comparative analysis between RAGE knockout and control littermate‐derived primary neuronal cultures. F,I) Evaluation of pathological changes in 5xFAD mice brain after 30 days of oral 6‐TG treatment, exhibiting Alzheimer's pathology‐related protein expression, including the STAT1‐JAK2 pathway and its downstream target BACE1, using immunoblot analysis. G) Immunofluorescence imaging for Aβ plaques (green) and microglial marker Iba1 (red) in the hippocampus of 5xFAD mice, imaged under a 10× objective. Scale bar = 80 µm. H) Quantitative analysis of the volume and distribution of the top 60 Aβ plaques in each sample, employing Imaris software for image analysis. Comparison between vehicle‐treated (red) and 6‐TG‐treated (0.5mg kg^−1^) (green) groups. Data are presented as mean (SD). Statistical significance was derived from unpaired t test relative to vehicle treated controls.

To validate the hypothesis that RAGE is a primary target of 6‐TG, as suggested by our transcriptomic analysis and predictions from MT‐DTI model, we first investigated the direct interaction between 6‐TG and RAGE. Utilizing surface plasmon resonance (SPR), we quantitatively measured this binding, obtaining consistent evidence of 6‐TG affinity for RAGE, with dissociation constant (*K*_d_) values ranging from 400 nm to 16.9 µm (**Figure** **6**A; and Figure S6, Supporting Information), demonstrating a significant binding affinity (representative *K*_d_ = 400 nm). These results support our drug‐target interaction predictions, despite the actual binding affinity being lower than anticipated. Further validation was achieved through a fluorescence resonance energy transfer (FRET) assay, which confirmed that 6‐TG effectively disrupts the formation of the RAGE‐Aβ complex, as evidenced by a decrease in FRET efficiency (Figure S7A–C, Supporting Information). This indicates competitive binding by 6‐TG to RAGE, thus inhibiting the RAGE‐mediated adverse effects. To further substantiate the effect of 6‐TG on the Aβ‐RAGE axis, we conducted Thioflavin T fluorescence assays to evaluate its impact on Aβ aggregation. The results revealed no significant changes in Aβ aggregation under 6‐TG treatment conditions (Figure S7D, Supporting Information). This suggests that 6‐TG primarily modulates the Aβ‐RAGE axis by binding to RAGE, rather than directly influencing Aβ aggregation.

Cellular experiments further clarified the involvement of RAGE in modulation of BACE1 expression with 6‐TG. Contrary to the expected upregulation of BACE1 upon RAGE pathway activation,^[^ ^22^ ^]^ our findings revealed that RAGE overexpression in SH‐SY5Y cells did not increase BACE1 levels. Notably, 6‐TG treatment led to a more significant reduction in BACE1 levels in RAGE‐overexpressing cells compared to control cells (Figure 6B,C). Conversely, silencing RAGE expression negated the BACE1‐lowering effect of 6‐TG (Figure 6D), establishing RAGE's crucial role in the action mechanism of 6‐TG. This was further supported by experiments in primary neurons from RAGE knockout mice, which showed a loss of the 6‐TG‐mediated BACE1 reduction (Figure 6E), affirming the essential function of RAGE in the 6‐TG mechanism.

Pathological assessments in 5xFAD mice treated with 6‐TG revealed alterations in AD‐related protein expression and a reduction in Aβ plaques, although no significant changes were observed in Aβ levels within the cerebrospinal fluid (CSF, Figure 6F–H; and Figure S7E, Supporting Information). The STAT1‐JAK2 signaling pathway, associated with neuroinflammation and neurodegeneration,^[^ ^29^ ^]^ was downregulated following 6‐TG treatment (Figure 6I), correlating with alterations in BACE1 expression and suggesting a neuroprotective effect of 6‐TG.

Collectively, these findings underscore the potential of 6‐TG as a modulator of the RAGE‐BACE1 axis, offering mechanistic insights into its therapeutic efficacy in AD.

### 2.7 6‐TG Enhances Microglial Phagocytosis and Modulates Soluble RAGE Levels

**Figure 7:**
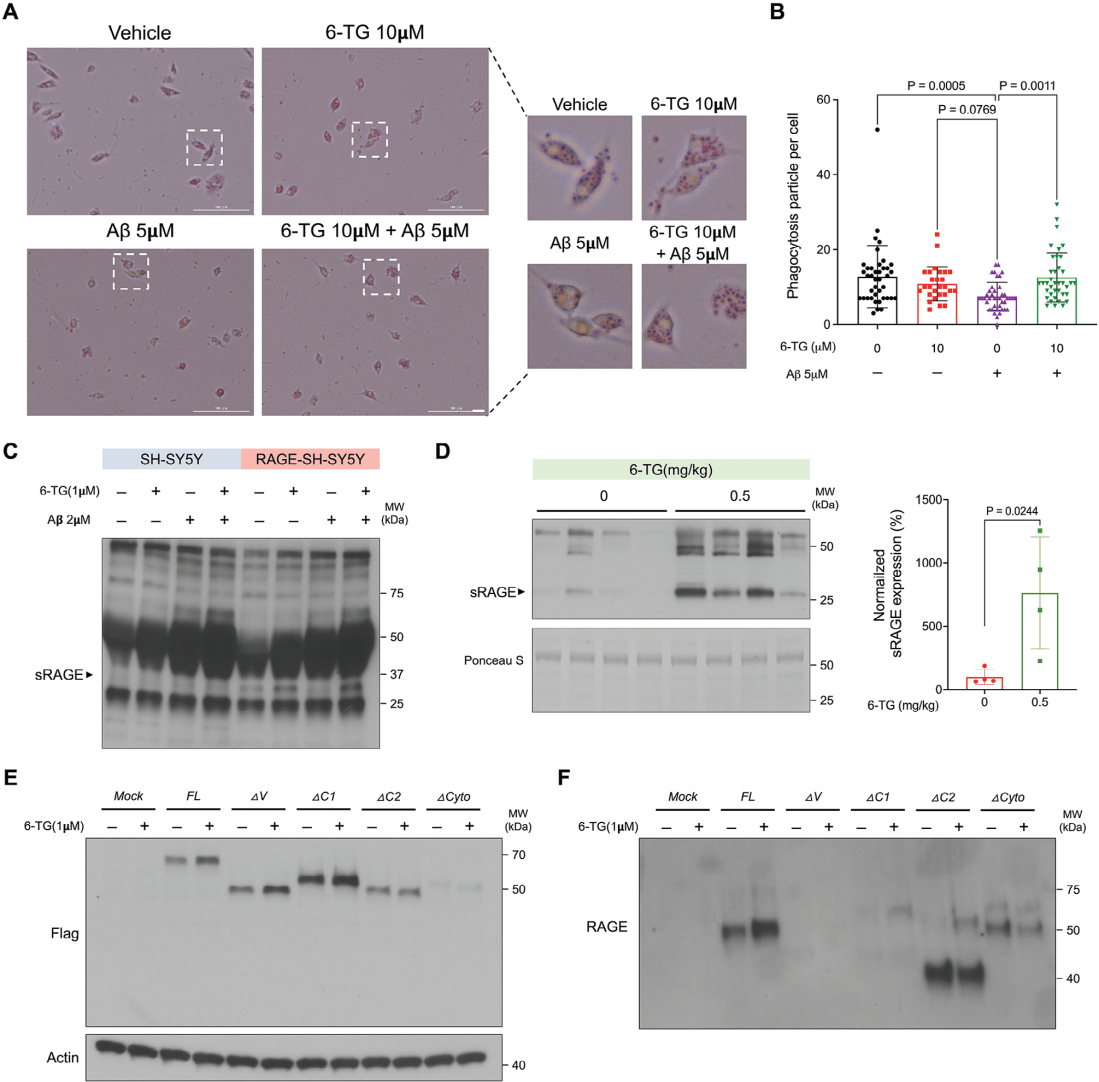
6‐TG modulation of microglial phagocytosis and RAGE dynamics. A) Enhanced phagocytic activity in primary mouse microglia treated with 6‐TG, demonstrated by micrographs capturing pH‐sensitive Zymosan BioParticles uptake. Insets magnify the area of increased particle internalization, evidenced by intensified red fluorescence within phagolysosomes, following 6‐TG treatment to counteract Aβ‐impaired phagocytic function. Scale bar = 20 µm. B) Quantitative analysis of phagocytic activity across triplicate assays. Data are presented as mean (SD) with *P* values derived from ordinary one‐way ANOVA and Holm–Šídák's multiple comparisons test, denoting statistical significance relative to Aβ only treated controls. C) sRAGE in the culture media of neuronal cells post 6‐TG or Aβ treatment, reflecting RAGE expression modulation impact. D) Increased sRAGE in the cerebrospinal fluid of C57BL/6 mice after oral administration of 6‐TG, demonstrated by blots reflecting sRAGE level changes consequent to a 12‐day 6‐TG regimen. Data are presented as mean (SD). Statistical significance was derived from unpaired *t*‐test relative to vehicle treated controls. E,F) 6‐TG effects on RAGE variant expression, depicted in representative blots for HEK293 cell lysates E) and culture media F), post‐transfection with various RAGE constructs and 6‐TG treatment.

We demonstrated a reduction in neuroinflammatory markers in 6‐TG‐treated mice, alongside a notable expression of RAGE proteins in microglia. This prompted an investigation into whether 6‐TG could enhance microglial phagocytosis, a critical function impaired in AD pathophysiology, especially under Aβ exposure. Using pH‐sensitive Zymosan BioParticles to culture primary mouse microglia, we observed that 6‐TG treatment significantly enhanced phagocytosis, counteracting the Aβ‐induced impairment (**Figure** **7**A). A quantitative assessment confirmed the substantial improvement in phagocytic activity in 6‐TG treated cells compared to those exposed solely to Aβ, highlighting the ability of 6‐TG to alleviate Aβ‐related microglial dysfunction (Figure 7B). Importantly, this beneficial effect of 6‐TG on phagocytosis was absent in microglia lacking RAGE (Figure S7F,G, Supporting Information), emphasizing the crucial role of RAGE in this mechanism.

In 5xFAD mouse brain tissues and SH‐SY5Y neuroblastoma cells, sRAGE levels increased following 6‐TG treatment (Figure 6B,C,F,C; and Figure S8A,C, Supporting Information). To directly link this increase to 6‐TG, we administered the compound orally to C57BL/6 mice over 12 days (Figure S8D, Supporting Information) and measured sRAGE levels in CSF (Figure 7D) and plasma (Figure S8E, Supporting Information). Results showed a significant elevation in sRAGE, affirming the role of 6‐TG in enhancing RAGE cleavage. To confirm the specific effects of 6‐TG, we assessed its plasma absorption and ability to penetrate the BBB using liquid chromatography (LC) following oral administration. Detectable levels of 6‐TG in both plasma (0.12 ppm) and brain tissues (0.1 ppm) demonstrated both effective absorption and BBB penetration (Figure S8F–H, Supporting Information).

Further exploration into the specific RAGE domain involved in 6‐TG‐induced cleavage utilized RAGE domain deletion constructs in HEK293 cells. Treatment with 6‐TG led to varied sRAGE levels in the culture media, depending on the presence of specific RAGE domains (Figure S8I, Supporting Information). Cells expressing full‐length RAGE or ∆C1 construct showed increased sRAGE levels post‐treatment, while those with ∆C2 and ∆Cyto constructs showed decreased or undetectable sRAGE. Notably, no sRAGE was observed in the ∆*V* construct. These findings suggest that the C2 domain contributes to the cleavage of RAGE to sRAGE, while the ∆*V* domain appears to be indispensable for this conversion process (Figure 7E,F).

To further elucidate the binding process and potential binding sites of 6‐TG, molecular docking simulations were conducted. The simulations identified a probable cross‐attention binding site for 6‐TG within the V domain of RAGE, which is a critical binding site for various ligands, including Aβ and AGEs. The docking results revealed a high degree of concordance between the predicted key residues in the V domain and the anticipated cross‐attention binding site (Figure S9, Supporting Information). Detailed docking analysis highlighted two primary binding sites, designated as Sites 1 and 2. Docking scores were computed using the PDB structure of the V domain, with Site 1 showing the highest docking score of 6‐TG (Figure S10 and Table S1, Supporting Information). Furthermore, compounds with structural similarity to 6‐TG also showed high predicted docking scores, further supporting the binding potential of 6‐TG to the RAGE‐V domain. This docking score diminished when docking was conducted with the C1 domain. Analysis of the docking pose revealed an expected interaction when the purine moiety of 6‐TG overlapped like as the indole scaffold of the other ligand which is bound to RAGE V domain, particularly noted at K110 and K52 through Pi‐cation interactions. Integrating the results from simulation analyses with those obtained using the RAGE domain deletion constructs (Figure 7E,F) suggests that the V domain of RAGE plays a crucial role in the 6‐TG mechanism. This evidence underlines the importance of precise molecular interactions within the RAGE V domain, highlighting its significance in therapeutic target identification and the development of intervention strategies against AD mediated by RAGE interactions.

## 3 Discussion

AD, the leading cause of dementia, is witnessing a surge in prevalence with the aging global population. Despite this increase, a definitive cure for AD remains out of reach. Current treatments, such as Donepezil, provide only symptomatic relief without halting disease progression. The accumulation of Aβ plaques, a key pathological feature of AD, is primarily governed by the enzyme BACE1, identifying it as a pivotal target for interventions.^[^ ^14^, ^30^ ^]^ Despite the development of numerous BACE1 inhibitors, including Verubecestat (MK‐8931)^[^ ^31^ ^]^ and Lanabecestat (AZD3293),^[^ ^32^ ^]^ have shown initial promise by reducing Aβ levels, their efficacy in slowing cognitive decline has not been proven in later‐stage clinical trials, leading to their discontinuation.^[^ ^33^ ^]^ The broad physiological roles of BACE1, encompassing myelination,^[^ ^16^, ^17^ ^]^ synaptic functionality,^[^ ^16b^ ^]^ membrane protein regulation,^[^ ^34^ ^]^ and immunomodulation,^[^ ^35^ ^]^ complicate the therapeutic targeting of BACE1 due to potential side effects associated with its inhibition.^[^ ^36^ ^]^

Addressing these limitations, our research focused on modulating BACE1 expression rather than inhibiting its enzymatic function. This strategy stems from the understanding that even minor increases in BACE1 expression can precipitate the pathological accumulation of Aβ.^[^ ^14a^ ^]^ Evidence supports heightened BACE1 expression in Alzheimer's pathology, underscoring the enzyme's dual role in disease progression^[^ ^12^, ^37^ ^]^ and normal brain function, including synaptic plasticity and neurodevelopment.^[^ ^38^ ^]^ The advanced approach of downregulating BACE1 expression, compared to direct inhibition, aims to mitigate amyloidogenic processes while preserving the physiological functions of BACE1, thereby offering a more balanced therapeutic strategy.

Employing a BACE1 promoter‐based screening system, we identified 6‐TG as a compound with potential efficacy in downregulating BACE1 expression. Through in vitro and in vivo assays, including promoter deactivation and GFP reporter assays, 6‐TG demonstrated a capacity to diminish BACE1 expression and Aβ production, suggesting a restorative effect on synaptic density and cognitive functions in AD models. Extending these observations to hESC‐derived neurons, GO analysis revealed a significant enrichment of genes associated with synaptic formation, indicating that 6‐TG may exert a profound effect on synaptogenesis. This synaptic influence aligns with the observed cognitive improvements, positioning 6‐TG as a modulator of synaptic integrity and function in the context of neurodegenerative disease. Consistent with these findings, the GO results obtained from mouse neurons under Aβ‐induced toxicity conditions also demonstrated that 6‐TG treatment was associated with increased calcium signaling, stabilization of the cytoskeleton, and enhanced axon guidance. This indicates that 6‐TG plays a protective role in mitigating Aβ toxicity and preserving neuronal function. To determine how 6‐TG influences BACE1 expression, we conducted in vitro and in vivo studies. Our findings suggest that the impact of 6‐TG on BACE1 expression represents an off‐target effect, diverging from its primary pharmacological action. As an anticancer agent, the main mechanism of 6‐TG involves DNA binding to inhibit cell proliferation.^[^ ^26^, ^39^ ^]^ This primary action could not explain the reduction in BACE1 expression observed in our experiments. The discrepancy between its known mechanism of inhibiting cell proliferation and the modulation of BACE1 expression indicates an indirect relationship, especially since the dosage used in our studies (0.5 mg kg^−1^) was significantly lower than the effective dose (8.1 mg kg^−1^)^[^ ^40^ ^]^ required for its canonical anticancer activity. Additionally, promoter and cell viability assays, which confirmed the absence of apoptosis, support the conclusion that the conventional mechanism of 6‐TG does not underlie the observed effects on BACE1 expression.

To elucidate the molecular mechanisms underlying 6‐TG modulation of BACE1 expression, we integrated next‐generation sequencing analysis with a structure‐based algorithm designed to predict drug‐protein interactions. This multifaceted approach successfully pinpointed RAGE as a tentative binding partner for 6‐TG. SPR assays were subsequently conducted to empirically validate the predicted interaction between RAGE and 6‐TG, revealing a definitive binding affinity that corroborates our computational predictions. This empirical data lends substantial support to the hypothesized regulatory role of RAGE in the mechanism by which 6‐TG influences BACE1 expression.

The investigation into the signaling pathway of RAGE unveiled a significant correlation with BACE1 expression. Prior research has illustrated that RAGE activation can catalyze an upsurge in BACE1 expression and activity,^[^ ^22^, ^41^ ^]^ implicating RAGE signaling pathways, notably the NF‐κB pathway, in the transcriptional regulation of BACE1 and subsequent Aβ production.^[22b, 41b]^ Additionally, RAGE activation is known to engage various MAPK pathways^[^ ^42^ ^]—^including extracellular signal‐regulated kinase (ERK), c‐Jun N‐terminal kinase (JNK), and p38 MAPK—linked to cellular processes such as proliferation, differentiation, inflammation, and stress response, with the JNK pathway and its principal transcription factor, activator protein‐1 (AP‐1), playing a crucial role in BACE1 regulation. Furthermore, RAGE signaling can activate the (STAT) pathway.^[24a, 29b, 29c]^ Indeed, several studies have provided evidence supporting the involvement of STAT1 and STAT3 in the upregulation of BACE1 expression at the molecular level.^[^ ^43^ ^]^ Remarkably, our research findings align closely with the outcomes of the prior reports showing that these transcription factors have been implicated in the regulation of BACE1 expression.

Our comprehensive analysis, employing sequential BACE1 promoter deletion and sequence analysis, revealed that 6‐TG specifically impact a particular promoter region (−1150 ≈−815, Figure S1A–E, Supporting Information), influencing the activity of key transcription factors involved in BACE1 regulation, such as NF‐κB, HIF1α, YY1, Sp1, STAT3, and STAT1. Experimental data from the 5xFAD AD animal model showed that 6‐TG administration leads to a marked reduction in the levels of pJAK2 and pSTAT1, suggesting that 6‐TG exerts its regulatory effect on BACE1 expression through the modulation of the STAT1 pathway.

Additionally, the reduction in pJAK2‐pSTAT1 pathway activity induced by 6‐TG appears to be associated with an elevation in sRAGE levels. The amount of sRAGE increased in both in vitro and in vivo models following 6‐TG treatment. The augmented sRAGE levels could signify two significant implications. First, sRAGE is generated through the cleavage by enzymes, such as MMP9^[^ ^42^ ^]^ and ADAM10.^[^ ^44^ ^]^ This process can inhibit the activation of the RAGE‐pJAK2‐pSTAT1‐BACE1 pathway, thereby alleviating the neuroinflammatory function of RAGE. Second, the increased sRAGE can sequester Aβ, counteracting the potential interaction between RAGE and Aβ.

The utility of docking prediction analysis simulations in this study has provided substantial insights into the molecular mechanisms underlying the interaction of 6‐TG with the RAGE. Our findings underscore the efficacy of these simulations in elucidating the interaction dynamics between small molecules and their protein targets, particularly in complex biological systems implicated in AD. Notably, the alignment of key residues within the V domain, which are predicted cross‐attention sites, enhances our understanding of the structural basis of 6‐TG binding affinity. Detailed characterizations of docking poses provide critical insights into the molecular interactions that underlie 6‐TG efficacy and RAGE activity. Integrating these computational predictions with experimental data from RAGE domain deletion constructs further solidifies the central role of the RAGE V domain in the therapeutic mechanism of 6‐TG. By bridging computational predictions with empirical evidence, our study provides profound insights into the action mechanisms of 6‐TG.

While our findings provide valuable insights into the therapeutic potential of 6‐TG for AD via its interaction with the RAGE, several limitations necessitate further investigation. First, although we have predicted the binding of 6‐TG to RAGE and its subsequent conversion to sRAGE, these mechanisms have not been conclusively proven. Furthermore, while expected binding sites on RAGE have been identified through computational methods, experimental validation of these sites remains incomplete. The variations in sRAGE levels observed when employing different RAGE domain deletion models reveal a complex relationship between the conversion site and binding site of RAGE with 6‐TG. This discrepancy underscores the necessity for further research to understand the mechanisms underlying the RAGE therapeutic in AD treatment. Finally, the doses of 6‐TG administered to AD model mice were lower than those used clinically, raising questions about whether similar neuroprotective effects can be achieved at clinically relevant doses.

The RAGE signaling pathway, a pivotal inducer of neuroinflammatory responses in AD, has been extensively targeted, leading to the development of small molecule antagonists designed to inhibit its activity.^[^ ^45^ ^]^ Concurrently, employing sRAGE as a decoy receptor has emerged as a strategy to mitigate the effect of the RAGE pathway.^[^ ^23^, ^46^ ^]^ However, a principal challenge with recombinant sRAGE‐based therapies is their limited capacity to traverse the BBB,^[^ ^46a^ ^]^ a critical barrier for achieving neuroprotective outcomes in the brain. Unlike the constraints associated with sRAGE recombinant protein therapies, 6‐TG offers a distinct advantage by intracerebrally modulating sRAGE levels. This unique property of 6‐TG, enhancing sRAGE concentrations within the brain, introduces a novel action mechanism that bypasses the hurdles of direct sRAGE administration. Furthermore, its direct interaction with RAGE inhibits the detrimental effects of the pathway. This dual functionality not only underscores the potential of 6‐TG for AD therapy repurposing but also catalyzes further exploration into leveraging sRAGE modulation as a therapeutic avenue.

## 4 Conclusion

In conclusion, our results demonstrate that regulating BACE1 expression can effectively mitigate AD, highlighting the therapeutic potential of targeting the RAGE‐BACE1 pathway with RAGE modulator. RAGE plays an influential role in AD by binding to Aβ proteins, promoting inflammatory responses, and increasing neurotoxicity. Furthermore, RAGE upregulates the expression of BACE1 enzyme, leading to increased Aβ production and accelerating the progression of AD. The study reveals that the anticancer drug 6‐TG not only reduces BACE1 expression and decreases Aβ accumulation but also improves cognitive function in an AD mouse model and enhances the phagocytic activity of microglia. It directly interacts with RAGE, modulating BACE1 expression via the JAK2‐STAT1 pathway and increasing sRAGE levels in the brain. These findings suggest a novel approach to AD therapies by modulating the RAGE‐BACE1 axis and support small molecule strategies to increase brain sRAGE levels, offering a strategic alternative to address the complexity of AD.

## 5 Experimental Section

### Cell Line and Primary Neuron Cell Culture

Cell lines: Human neuroblastoma SH‐SY5Y cells, APP‐SH‐SY5Y cells, RAGE‐overexpressing SH‐SY5Y cells, hB1PG‐RFP SH‐SY5Y cells, and HEK293T cells (transfected with RAGE constructs, as shown in Figure S8F, Supporting Information) were maintained in Dulbecco's modified Eagle medium (DMEM, Hyclone/ SH30243.01) supplemented with 10% fetal bovine serum (FBS, Hyclone/ SH30084.03) and 1% penicillin‐streptomycin (PS, Gibco/ 15140122). Cell cultures were incubated at 37 °C in a 5% CO_2_ atmosphere. SH‐SY5Y cell line (Wild type, hB1PG‐RFP SH‐SY5Y) which were cultured in DMEM with 10% FBS, 1%PS, and 1 µg mL^−1^ puromycin. *hB1PG‐RFP SH‐SY5Y: The pHB1PG plasmid (Figure S1B, Supporting Information) was then transfected into SH‐SY5Y cells, a neuronal cell line, along with a pcDNA3.1‐mCherry vector (Figure S1C, Supporting Information) to determine the cell count, resulting in a stable cell line (hB1PG‐RFP SH‐SY5Y) expressing both red fluorescent protein (RFP) and green fluorescence protein (GFP). Primary neuronal cultures: Primary cortical and hippocampal neurons were isolated from embryonic day 17 C57BL/6 and RAGE knockout mice. Tissue dissection and dissociation into single‐cell suspensions were performed as described previously^[^ ^47^ ^]^ with slight modifications. Single cells were plated at a density of 2 × 10^6^ cells per well in six‐well plates and cultured in DMEM supplemented with 10% FBS and 1% PS for 1 day. The medium was then replaced with Neurobasal medium (Gibco/21103049) containing B27 supplement (Gibco/A3582801) and 1% PS to inhibit glial proliferation. Primary neurons were cultured for 14 days with media changes every 2 days before pharmacological treatments.

### Quantitative RT‐PCR Analysis

Total RNA was extracted from SH‐SY5Y cells and primary neuronal cultures post 24 h treatment with 6‐TG, utilizing TRIzol reagent (Invitrogen/15596026). Mouse brain tissues were segmented into cortical and hippocampal regions, each weighing ≈25 mg, for subsequent RNA isolation. Purified RNA (1 µg) was reverse transcribed using the cDNA synthesis kit (TaKaRa/ RR047A) on a T100 Thermal Cycler. Quantitative real‐time PCR amplification was conducted using TB Green Premix Ex Taq II (TaKaRa/ RR820A) on a CFX96 Real‐Time System (Bio‐Rad). Gene‐specific primers (as listed in Table S2, Supporting Information) were employed for the amplification of target transcripts. The relative expression levels were normalized to housekeeping genes and calculated using the 2^−ΔΔCt^ method.

### Western Blotting

Sample harvesting and preparation: In vitro cell lysates were prepared using protease and phosphatase inhibitor cocktail (Thermo Fisher Scientific/78444) in T‐PER tissue protein extraction buffer (Thermo Fisher Scientific/78510). In vivo, proteins were extracted from the cortex and hippocampus of mouse brains using a homogenizer in T‐PER buffer supplemented with the inhibitor cocktails. The samples were then subjected to high‐speed centrifugation at 13 000 rpm for 10 min. The supernatant concentration was quantitatively determined using the BCA assay. SDS‐PAGE and Western Blotting: Protein samples (10–20 µg), prepared with 4× sample buffer, were denatured by boiling at 95 °C for 10 min. The denatured proteins were resolved on 8%–15% SDS‐PAGE gels and subsequently transferred to 0.45 µm PVDF membranes (Merck/ IPVH00010) at 80–100 V and 200 mA for 70–100 min. The membranes were blocked with 5% v/v skim milk in Tris‐buffered saline (pH 7.5) containing 0.2% Tween 20 (TBS‐T) for 1–3 h. Immunoblotting: The blocked membranes were incubated with primary antibodies overnight at 4 °C (refer to Table S3 (Supporting Information) for antibody details). This was followed by incubation with HRP‐conjugated secondary antibodies. Immunoreactive bands were visualized using the chemiluminescent HRP substrate kit (Cytiva/ RPN2106).

### Promoter Assay

HEK293T cells were seeded at a density of 7.5 × 10^4^ cells per well in 24‐well plates and allowed to adhere for 24 h. Cells were then transfected with 200 ng of the pB1PA plasmid harboring the BACE1 promoter and 5 ng of the pRTK vector as a transfection control. Following a 4 h incubation post‐transfection, cells were treated with 6‐TG across a range of concentrations. After a 20 h incubation period with 6‐TG, cells were washed once with 1X PBS. Luciferase activities, both Firefly and Renilla, were quantified using the Dual‐Luciferase Reporter Assay System in accordance with the instructions provided by the manufacturer (Promega/E1910).

### RNA Isolation and Quality Assessment

Total RNA was isolated from mouse primary neurons and human embryonic stem cell‐derived neurons employing TRIzol reagent. RNA integrity and quality were verified using the HWI‐ST1276 platform (Novogene), and samples exhibiting BACE1 expression surpassing that of vehicle‐treated controls were selected for subsequent sequencing.

### Library Construction and Sequencing

Libraries for RNA sequencing were prepared according to Eukaryotic RNA‐Seq library preparation protocol of Novogene. Sequencing was performed on the NovaSeq PE150 system, generating 150‐bp paired‐end reads with a minimum yield of 6 GB per sample.

### Oligomeric Aβ

Preparation of Aβ Peptide: Dissolve 1 mg of Aβ peptide in 2 mL of 1,1,1,3,3,3‐hexafluoroisopropanol (HFIP) in a sealed container. As HFIP is highly toxic, perform this step under a chemical fume hood. Shake the solution daily and allow it to incubate at room temperature for 3 days. After incubation, aliquot 100 µL of the solution into 20 tubes, resulting in ≈50 µg of peptide per tube. Verify the peptide concentration by measuring the protein content. If necessary, prepare aliquots at a higher concentration. Completely evaporate the HFIP from the aliquots using a SpeedVac vacuum concentrator. Store the aliquots at −70 °C, wrapping each tube in parafilm for preservation until use. Preparation of Aβ Oligomers: Dissolve the prepared Aβ peptide in anhydrous dimethyl sulfoxide (DMSO, 11 µL) to a concentration of 1 mm. Vortex the solution for 1 min and centrifuge briefly. Allow the solution to stabilize at room temperature for 10 min. Dilute the peptide in cold F‐12 media (add 99 µL media) to achieve a final concentration of 100 µm. Vortex the solution vigorously for 30 s. During this step and all subsequent steps, keep the sample on ice. Incubate the solution at 4 °C for 24 h to promote oligomer formation. Use the oligomers immediately after preparation.

### Animals and 6‐TG Treatment

Hemizygous APP/PS1 (APPswe/PSEN1dE9) double‐transgenic mice were bred and maintained on a C57BL/6J genetic background. The animals were housed under controlled conditions, observing a 12 h light‐dark cycle, with ad libitum access to food and water. At 6 months of age, male APP/PS1 mice and their nontransgenic littermates were randomly allocated into four groups: nontransgenic littermates (*n* = 7), vehicle‐treated (1:500 DMSO:water, *n* = 8), and two groups receiving 0.1 mg kg^−1^ (*n* = 7) and 0.5 mg kg^−1^ (*n* = 8) of 6‐thioguanosine via oral gavage, administered daily for 38 consecutive days.

Behavioral assessments commenced following 30 days of treatment. Mice's body weight was monitored daily to evaluate potential toxic effects of 6‐TG. The Morris water maze protocol initiated on day 31, involving daily training sessions until day 34. Long‐term memory retention was assessed on day 35 via probe trials. On day 37, mice were conditioned for the passive avoidance test, with the actual test conducted on day 38.

Subsequent to the behavioral tests, mice were euthanized on day 38; anesthesia was induced with Zoletil (1 mL kg^−1^), followed by perfusion with phosphate‐buffered saline (PBS, 0.9% NaCl). Brain tissues were excised immediately postperfusion; a portion was flash‐frozen in liquid nitrogen and stored at −70 °C, and the remainder was fixed in 4% paraformaldehyde for 24 h, then cryoprotected in 30% sucrose at 4 °C in preparation for immunohistochemical analysis.

The experimental protocol, including animal treatment and care, was approved by the Institutional Animal Care and Use Committee (IACUC) of Sungkyunkwan University (SKKUIACUC‐20150041), ensuring adherence to ethical guidelines. All procedures were designed to minimize animal suffering and the number of animals utilized.

### H&E Staining

Harvested tissues, including the heart, kidney, liver, lung, and spleen, were fixed in 4% paraformaldehyde at 4 °C for 48 h, followed by dehydration through a graded ethanol series, clearing in xylene, and embedding in paraffin. Tissue sections (5 µm thick) were prepared using a microtome and mounted on glass slides for hematoxylin and eosin (H&E) staining. The staining process involved deparaffinization in xylene, rehydration through descending concentrations of ethanol to distilled water, staining with hematoxylin (5 min), differentiation in 1% acid alcohol (2–3 s), and bluing in running tap water (2 min). Slides were counterstained with eosin Y (1–2 min), dehydrated through ascending ethanol concentrations, cleared in xylene, and mounted with a coverslip using a permanent mounting medium. Stained sections were examined and imaged using a Leica optical microscope (LEICA ICC50 E, LAS V4.13 software) to assess histological changes.

### Morris Water Maze Test

The spatial memory test was evaluated as previously described with minor modifications.^[^ ^3^ ^]^ The apparatus consisted of a white circular pool, 150 cm in diameter and 60 cm in height, with an unmarked inner surface. The pool was filled with water, maintained at 22±1 °C, and made opaque with nontoxic, water‐soluble white dye. It was segmented into four equal quadrants, with a translucent acrylic platform (9 cm diameter, 25 cm height) positioned in the center of one quadrant, submerged 1.5 cm beneath the surface, rendering it invisible at the water level. The pool was situated in a room surrounded by various stationary visual cues. A video tracking system equipped with ANY‐maze software was used to record each mouse's swimming path from start to finish. Over four consecutive days, the mice underwent four trial sessions daily, with a 1 h inter‐trial interval. The start position was altered for each trial, but the escape platform remained stationary throughout the experiment. Mice failing to find the platform within 60 s were guided to it, allowed to stay for 10 s, and then gently removed from the pool. For the probe trial, conducted 24 h following the final training session, the platform was removed to assess spatial memory retention over a 60 s trial period. During this test, latency was measured to the original platform location, time spent in the target quadrant, and the number of crossings over the platform's previous position.

### Passive Avoidance Test

The passive avoidance response was tested using a two‐compartment apparatus, comprising a lighted compartment and an adjacent dark compartment. On the training day, each mouse was placed in the illuminated compartment, facing away from the dark compartment, and allowed a 2 min acclimation period. Subsequently, the separating guillotine door was raised. The latency for the mouse to enter the dark compartment with all four paws was recorded, starting from the door opening. Upon full entry, the door was closed, and a foot shock (0.5 mA for 2 s) was administered after a 3 s delay. The mouse was then returned to its home cage following a 30 s postshock interval.

For the retention test conducted 24 h later, mice were again placed in the lighted compartment and the door was lifted after 2 min. The latency to enter the dark compartment was measured, with a maximum test duration of 5 min. Following the test, mice were returned to their home cages.

### Immunohistochemistry

Upon completion of behavioral assessments, fixed mouse brains were sectioned using a Leica CM3050S microtome into 30 µm slices. The sections were stored in a cryoprotectant solution comprising 80 mm K_2_HPO_4_, 20 mm KH_2_PO_4_, 154 mm NaCl, 300 g L^−1^ sucrose, 10 g L^−1^ polyvinylpyrrolidone, and 30% ethylene glycol.

### DAB Staining for Aβ Detection

Brain sections underwent quintuple 3 min washes in 1x PBS, followed by a 1 h block in a solution containing 5% fetal bovine serum and 0.1% Triton X‐100. They were then incubated overnight at 4 °C with primary antibodies (listed in Table S3, Supporting Information) within the blocking solution. DAB staining was subsequently executed following the provided kit protocol (Abcam/ ab64238).

### Immunofluorescence Protocol

The initial procedure mirrored that of DAB staining up to the blocking stage. After overnight incubation with primary antibodies (refer to Table S3, Supporting Information), slices were washed five times for 3 min each in 1x PBS. They were then incubated for 2 h with Alexa Fluor secondary antibodies, followed by three additional 3 min PBS washes. Sections were then mounted on glass slides using a DAPI containing mounting medium. Analysis focused on hippocampal regions CA1 and CA3, and the cerebral cortex, defined anatomically by the mouse brain atlas. Imaging was performed on a Zeiss LSM 710 META Duoscan confocal microscope with a 40× objective lens.

### ELISA

Brain tissue homogenization was carried out in T‐per extraction buffer supplemented with a protease inhibitor cocktail. The homogenates were then centrifuged at 13 000×g for 10 min at 4 °C, and the supernatants were collected for analysis. The concentrations of Aβ_40_ and Aβ_42_ peptides were quantified using a solid‐phase sandwich ELISA system (IBL International, Cat No. 27713 for Aβ_40_ and 27711 for Aβ_42_). Standards and brain extracts were incubated on a microtiter plate precoated with a monoclonal antibody specific to human Aβ (35–40) for Aβ_40_, or with an antibody to human Aβ (38–42) for Aβ_42_, at 4 °C overnight. Each well was washed with washing buffer for 7 times, the bound Aβ peptides were detected with HRP conjugated antihuman Aβ (11–28) and developed with TMB (3,3′, 5,5;‐tetramethylbenzidine) substrate in darkness at room temperature for 30 min. The enzymatic reaction was terminated with 1 N H_2_SO_4_, and absorbance was measured at 450 nm. Concentrations were determined by comparison with a standard curve.

### Fluorescence Resonance Energy Transfer (FRET) Assay

To delineate the interaction dynamics between the RAGE and 6‐TG, and to assess the inhibitory potential of 6‐TG or RAGE inhibitors, a FRET assay was performed. This method relies on energy transfer from a donor fluorophore (fluorescein isothiocyanate, FITC) to an acceptor fluorophore (Cyanine3, Cy3) when in proximity, indicative of molecular interactions.

Donor Label: Aβ_42_ peptide was labeled with FITC (Bachem/M‐2585), with a stock concentration of 100 µm in 100% DMSO. The working solution was diluted to a final concentration of 0.1 µm. Acceptor Label: Streptavidin‐conjugated Cy3 (Jackson Immuno Research/ 016‐160‐084), with a stock concentration of 1 mg/ml in 50% glycerol. The working solution was diluted to a final concentration of 2 µg mL^−1^. Linking: Biotinylated RAGE (EZ‐Link NHS‐PEG4 Biotinylation Kit, Thermo/ 21455), with a stock concentration of 1 mg mL^−1^. The working solution was diluted to a final concentration of 50 µg mL^−1^. Inhibition: The presence of 6‐TG or RAGE inhibitors was evaluated for their capacity to disrupt the RAGE‐Aβ interaction, as evidenced by changes in FRET efficiency. Prepare the reaction mixture by combining 50 µL of Cy3‐Streptavidin with 50 µL of biotinylated RAGE protein in PBS buffer. Incubate the reaction mixture at RT for 30 min with rotation to ensure thorough mixing. Dispense 100 µL of the Cy3‐RAGE mixture into each well of a 96‐well plate. Add 50 µL of the Aβ‐FITC solution to each well. Introduce 50 µL of the test sample solutions (6‐TG or RAGE inhibitors) to each well. Allow the reaction to proceed for 30–60 min at RT. Measure the fluorescence with the following settings: Excitation wavelength: 490 nm, Emission wavelength: 580 nm, shake duration before reading: 10 s. All measurements were taken at room temperature, with each condition tested in triplicate to ensure reproducibility. Data were analyzed with appropriate corrections for spectral overlap, direct excitation, and background fluorescence.

### Surface Plasmon Resonance (SPR)

SPR was employed to investigate the interaction kinetics between the RAGE and its ligands 6‐TG.

### Materials and Instruments

SPR Instrument: iMSPR‐Pro/A (icluebio, Korea). SPR Sensor Chip: CMD200 m (Carboxyl‐functionalized dextran surface, icluebio, Korea). Ligand: RAGE (Type1: Sino/ 11629‐HCCH/ RAGE_23‐351_, Type2: biovendor r&D/ RD172116100‐HEK/ RAGE_23‐347_ N‐Terminal His‐tag 14 AA) at 50 µg mL^−1^ in 5 mm Sodium acetate buffer, pH 5.0. Running Buffer: 1x HBST (100 mm HEPES, 150 mm NaCl, 0.005% Tween 20, pH 7.4). Analyte: 6‐TG.

### Procedure for Ligand Immobilization

Baseline Establishment: The system was equilibrated by passing 1x HBST at 30 µL min^−1^ until a stable baseline was achieved. Activation: The dextran surface of the sensor chip was activated with a 200 mm EDC/100 mm NHS solution at 30 µL min^−1^ for 5 min to facilitate covalent ligand attachment.

Washing: The surface was washed with 1x HBST at 30 µL /min^−1^ for 5 min to remove excess activating agents. Ligand Immobilization: RAGE was immobilized on the activated surface by injecting at 10 µL min^−1^ in 5 mm Sodium acetate buffer, pH 5.0, until reaching the desired response level.

Postimmobilization Wash: The surface was washed again with 1x HBST at 30 µL min^−1^ for 5 min to remove any unbound ligand. Blocking: Unreacted esters were deactivated using 1 m Ethanolamine at 30 µL min^−1^ for 3 min. Final Wash: The system was washed with 1x HBST at 30 µL min^−1^ until a stable baseline was re‐established postblocking.

### Procedure for Analyte Binding

Baseline: A stable baseline was established with 1x HBST at 30 µL min^−1^. Association: The TG compound analyte was injected at concentrations ranging from 0.1 to 0.15 µm in 1x HBST at 30 µL min^−1^ for 3 min to monitor binding kinetics. Dissociation: Postassociation, the dissociation of the analyte from RAGE was observed by continuing the flow of 1x HBST at 30 µL min^−1^ for 6 min. Data acquisition and kinetic analyses were performed using the iMSPR analysis software, applying a 1:1 binding model to fit the binding curves. Each experiment was conducted at room temperature, and all procedures were replicated to ensure reproducibility and accuracy of the results.

### Bioinformatics Analysis

Quality‐filtered sequencing reads were aligned to the reference genomes GRCm39 for mouse samples and GRCh38.p2 for human samples using the STAR aligner (v2.7.9a). Transcript assembly and quantification were performed using RSEM (v1.3.3). Differential gene expression analysis was conducted in R (v4.4.1) utilizing the DESeq2 package (v1.44.0) with a significance threshold set at *p* < 0.05. Data visualization and enrichment analyses were performed using a suite of R libraries and external tools. Principal component analysis (PCA) was carried out using the ggforce package, and volcano plots were generated with the EnhancedVolcano package. For gene ontology and pathway enrichment analyses, DAVID Bioinformatics Resources were used to interpret the biological significance of differentially expressed genes.

### Drug Library Screening Using Molecule Transformer‐Based Drug Target Interaction Model

To identify potential molecules within a chemical library, the Molecule Transformer‐based Drug Target Interaction (MT‐DTI) model was employed.^[^ ^28^ ^]^ This approach leverages the advanced capabilities of transformer models to analyze the complex molecular structures and predict their interactions with various protein targets.

### Liquid Chromatography

Plasma and brain samples were collected 30 min after oral administration of 6‐TG. Plasma was separated by centrifuging whole blood at 3000 × *g* for 10 min at 4 °C, and brain tissues were rapidly harvested and weighed. Plasma samples were prepared by diluting with 2 volumes of the mobile phase buffer (ACN/0.05 m KH₂PO₄ buffer [3%/97%, v/v] containing 0.005% H₃PO₄ with 5% DMSO), followed by vortexing for 10 s and centrifugation at 13 000 × *g* for 10 min at 4 °C to remove particulates. Brain tissues were homogenized in 3 volumes w/v of the same mobile phase buffer with 5% DMSO using a homogenizer, and the homogenates were centrifuged at 13 000 × *g* for 20 min at 4 °C. The resulting supernatants from both plasma and brain samples were filtered and subjected to LC analysis. A reversed‐phase C18 column (50 mm × 4.6 mm, 5 µm) was used for LC separation, with the mobile phase consisting of ACN/0.05 m KH₂PO₄ buffer (3%/97%, v/v) containing 0.005% H₃PO₄. The flow rate was set to 1.0 mL min^−1^, the injection volume was 10 µL, and the column temperature was maintained at 30 °C. Detection was performed at 341 nm. The LC analysis was conducted over a total runtime of 20 min. Calibration standards of 6‐TG prepared in the mobile phase buffer were used to construct a standard curve, and sample concentrations were calculated by comparing their peak areas to the calibration curve.

### Structural Modeling and Docking Analysis

For this study, the 6XQ5 PDB structure was employed and the Schrödinger suite (Maestro, version 13.1.141) for all procedures related to protein and ligand preparation as well as scoring assessments was utilized. The RAGE protein configuration was refined using the Protein Preparation Wizard from Schrödinger.^[^ ^48^ ^]^ Hydrogen bonds were determined through PROPKA at an assumed pH level of 7.0, drawing from empirical data to estimate p*K*a values and assign protonation states accordingly. Subsequently, a restrained minimization was conducted using the software's standard settings. Ligand configurations were protonated to reflect their likely states within a pH range of 7 ± 2.0. A prospective interaction site on the protein was predicted using a cross‐attention model,^[^ ^49^ ^]^ and receptor grids were generated accordingly. The Glide tool^[^ ^50^ ^]^ facilitated the docking of the protein and ligand.

### Aβ Plaque Size Analysis

Immunohistochemistry image analysis was conducted using Imaris software (Version 10.0.0, Bitplane Inc.). Confocal z‐stack images were initially imported into the Imaris software platform for processing. For the analysis of plaque, z‐stack images with a depth of 15 µm were acquired using Imaris. The volumetric densities of 6E10‐stained Aβ plaques were then calculated. Specifically, only plaques identified by 6E10 surfaces and with a volume top 60 samples were included in the analysis.

### Thioflavin T (ThT) Assay

The aggregation kinetics of Aβ were monitored in situ using a ThT fluorescence assay. Briefly, three independent samples were prepared for each experimental condition. Each sample contained ThT at a final concentration of 2.5 µm, in the presence or absence of 50 µm monomeric Aβ, and were incubated in PBS (pH 7.4) with 6‐TG at varying concentrations (1 nm to 10 µm). Samples were incubated in a 96‐well microplate at 37 °C with continuous shaking for 72 h. Fluorescence readings were taken every 12 h using a microplate reader with excitation and emission wavelengths set at 440 and 490 nm, respectively. To account for any background fluorescence from ThT alone, fluorescence values from samples without Aβ were subtracted from the corresponding readings of samples containing Aβ. This step minimized interference from the dye itself, which was determined to be negligible. The resulting corrected ThT fluorescence values were plotted as a function of time to evaluate Aβ aggregation kinetics. Statistical analysis was performed using Prism version 10, employing a repeated measures one‐way ANOVA method to analyze differences among experimental conditions.

### Statistical Analysis

Quantitative data were processed using Prism 10 (GraphPad Software). All Bar graphs presented in figures denote mean ± standard deviation (SD) derived from various measurements. Differences between two distinct experimental conditions were evaluated using unpaired *t*‐test for statistical significance. Comparison across multiple groups was performed using one‐way ANOVA, with posthoc analysis conducted via Dunnett's or Holm–Šídák's multiple comparisons test, as specified in the figure legends. Levels of significance are represented by numerical values. Sample sizes were not predetermined through statistical methods; instead, robustness was ensured by conducting multiple independent experiments with several replicates per sample.

## Conflict of Interest

H.Y., D.K., Y. Choi. and K.K. are employed by Deargen Inc., located in Daejeon, Republic of Korea. Although the company provides financial support for their employment, it does not directly benefit from the results of this study.

## Author Contributions

D.G.J. and S.H.B. designed the outline of the paper. D.G.J. and S.H.B. wrote the manuscript. S.H.B. performed most experiments in this study and prepared the figures. E.K. produced cloning and cell lines to build a screening system. Y.Y. executed screening assays for target drug selection. S.H. analyzed RAN‐seq data and cultured human embryonic stem cell‐derived neurons. S.H.B., S.H., J.P., and S.P. performed the experiments with the animals. Y.C. helped with the western bolt experiments and cell line maintenance. M.L. and E.K. performed the LC analysis. H.Y., D.K., Y.C., and K.K. were responsible for computational modeling analysis. S.J. supported patch‐clamp analysis. Y.K., Y.K.J., J.P.K., and S.W.S. supported RAGE related experiment. All authors participated in the review and editing of the manuscript.

## Supporting information



Supporting Information

## Data Availability

All data associated with this study are present in the paper or the Supporting Information with Data files. Additional data related to this paper may be requested from the authors.
