# Supplementary material for: A Novel RAGE Modulator Induces Soluble RAGE to Reduce BACE1 Expression in Alzheimer's Disease
Source: Adv Sci (Weinh). 2025 Jan 4;12(8):2407812. doi: 10.1002/advs.202407812 (PMC11848596; doi:10.1002/advs.202407812)
Supplement: Supplementary file 1 — Supporting Information [file ADVS-12-2407812-s001.docx]

**Suppporting Information**

**A Novel RAGE Modulator Induces Soluble RAGE to Reduce BACE1 Expression in Alzheimer's Disease**

Seung-Hyun Baek, Suji Hong, Eunae Kim, Sunyoung Park, Minyoung Lee, Jinsu Park, Yoonsuk Cho, Hyunjun Yoon, Daeseung Kim, Youngkwang Yun, Youbin Kim, Yoonjung Choi, Keunsoo Kang, Sangyong Jung, Jun Pyo Kim, Eunha Kim, Sang Won Seo, Yong-Keun Jung, and Dong-Gyu Jo^*^

*Correspondence should be addressed to: Dong-Gyu Jo

School of Pharmacy, Sungkyunkwan University, Suwon 16419, Republic of Korea

E-mail: jodg@skku.edu

**This Suppporting Information Includes:**

Figure S1. Mapping the active site using the human BACE1 promoter influenced by 6-TG.

Figure S2. Regulation of BACE1 expression by 6-TG and its structural analogues.

Figure S3. 6-TG preserves the intrinsic properties of primary neuronal cells.

Figure S4. Attenuation of AD pathology in the hippocampus of APP/PS1 mice by 6-TG.

Figure S5. Modulation of RAGE pathway-related gene expression by 6-TG in neuronal cells.

Figure S6. SPR analysis of 6-TG interaction with recombinant RAGE proteins.

Figure S7. Analyzing 6-TG interaction with RAGE and its impact on microglial phagocytosis.

Figure S8. sRAGE modulation by 6-TG in RAGE-overexpressing SH-SY5Y and in vivo.

Figure S9. Prediction of the binding site for RAGE-6-TG interaction.

Figure S10. Docking analysis of 6-TG interaction with RAGE.

Table S1. Binding energies of small molecules with the RAGE V-domain

Table S2. qRT-PCR primer information.

Table S3. Antibody information.


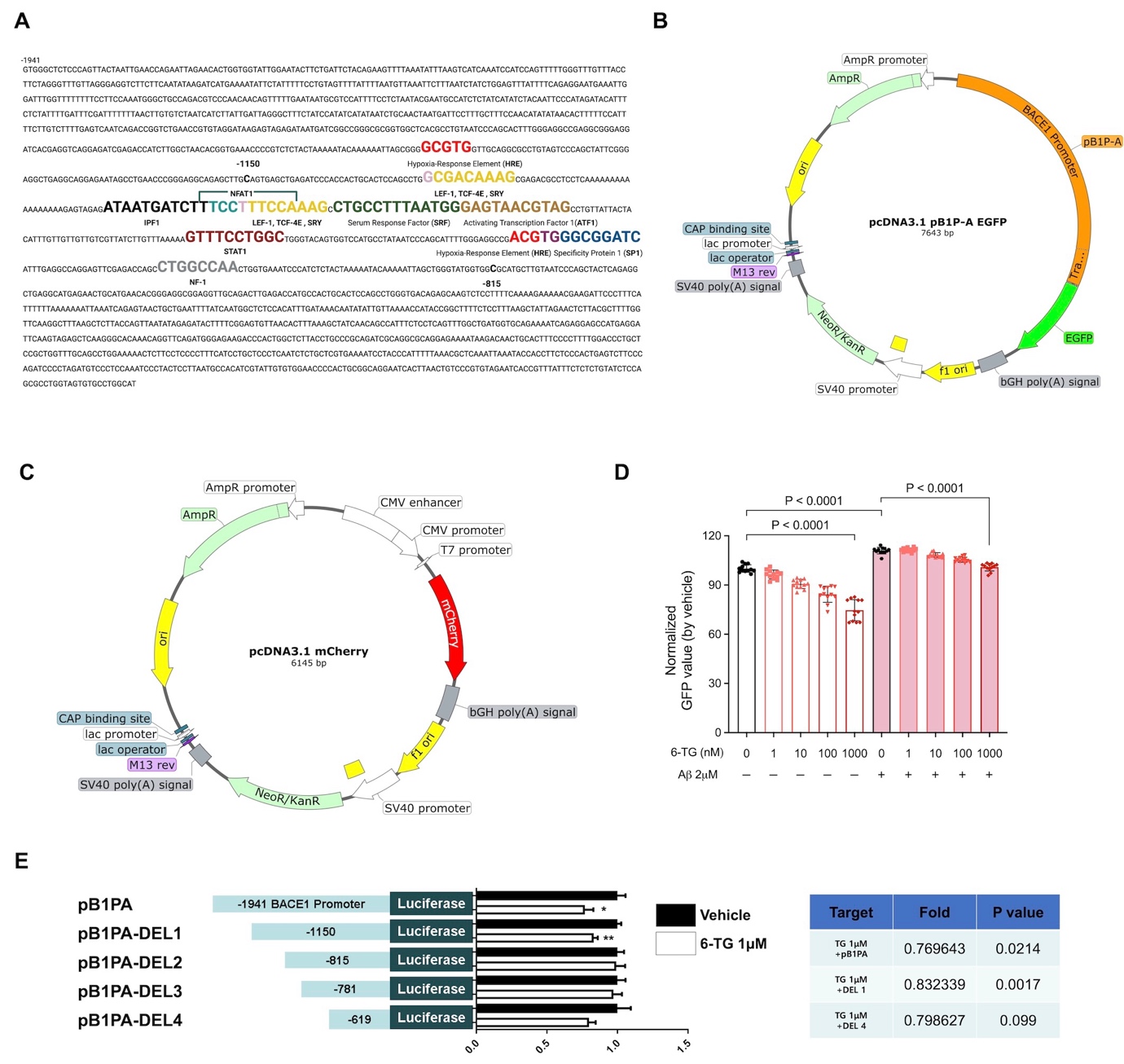


**Figure S1. Mapping the active site using the human BACE1 promoter influenced by 6-TG. (A)** Nucleotide sequence of the human BACE1 promoter, with annotation of potential transcription factor binding sites within the segment deleted from pB1PA-DEL1 to make pB1PA-DEL2. **(B and C)** Schematic representation of the plasmid constructs for creating the hB1PG-RFP SH-SY5Y cell line, illustrating the insertion sites and key genetic components of each construct. **(D)** Dose-response analysis of GFP signal intensity in hB1PG-RFP SH-SY5Y cells post 6-TG treatment with vehicle or Aβ, quantitatively assessed. Data are presented as mean (SD) with P values derived from ordinary one-way ANOVA and Dunnett’s multiple comparisons test, denoting statistical significance relative to Vehicle treated controls or Aβ only treated controls. **(E)** Evaluation of luciferase activity reflecting transcriptional dynamics in HEK293T cells transfected with pRLTK (5 ng) and pB1PA plasmids (200 ng), followed by 6-TG or vehicle treatment. The resultant luciferase expression provides insights into the transcriptional changes of BACE1 elicited by 6-TG. Data are presented as mean (SD). *P < 0.05; and **P < 0.01.


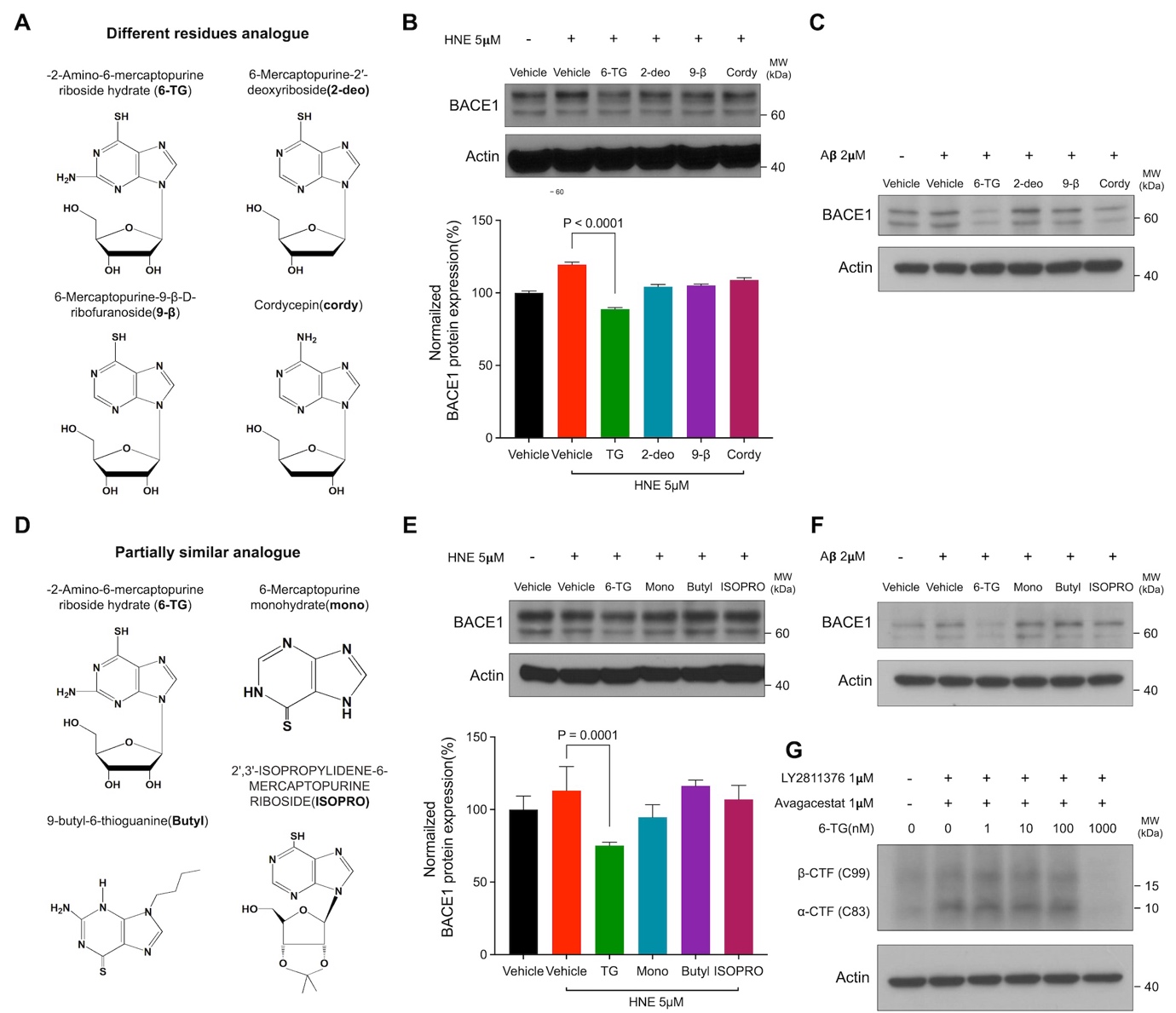


**Figure S2. Regulation of BACE1 expression by 6-TG and its structural analogues. (A)** Chemical structures of 6-TG and its distinct residue analogues. **(B, C, E and F)** SH-SY5Y cells were pretreated with 5 μM HNE (B and E) or 2 μM Aβ (C and F) to induce BACE1 expression before being exposed to 6-TG (1 μM) and its analogues (1 μM). BACE1 protein quantification was normalized to β-actin levels. **(D)** Chemical structures of 6-TG and its partially similar analogues. **(G)** Evaluation of CTF-α levels in APP-SH-SY5Y cells treated with varying concentrations of 6-TG under conditions of inhibited amyloidogenic processing. Cells were pre-treated with 1 µM LY2811376 (β-secretase inhibitor) and 1 µM Avagacestat (γ-secretase inhibitor) to suppress amyloidogenic pathways before the addition of 6-TG. No significant increase in CTF-α levels was observed. 1 µM 6-TG treatment resulted in cytotoxicity in the cells. Data are presented as mean (SD) with P values derived from ordinary one-way ANOVA and Dunnett’s multiple comparisons test, denoting statistical significance relative to HNE only treated controls.


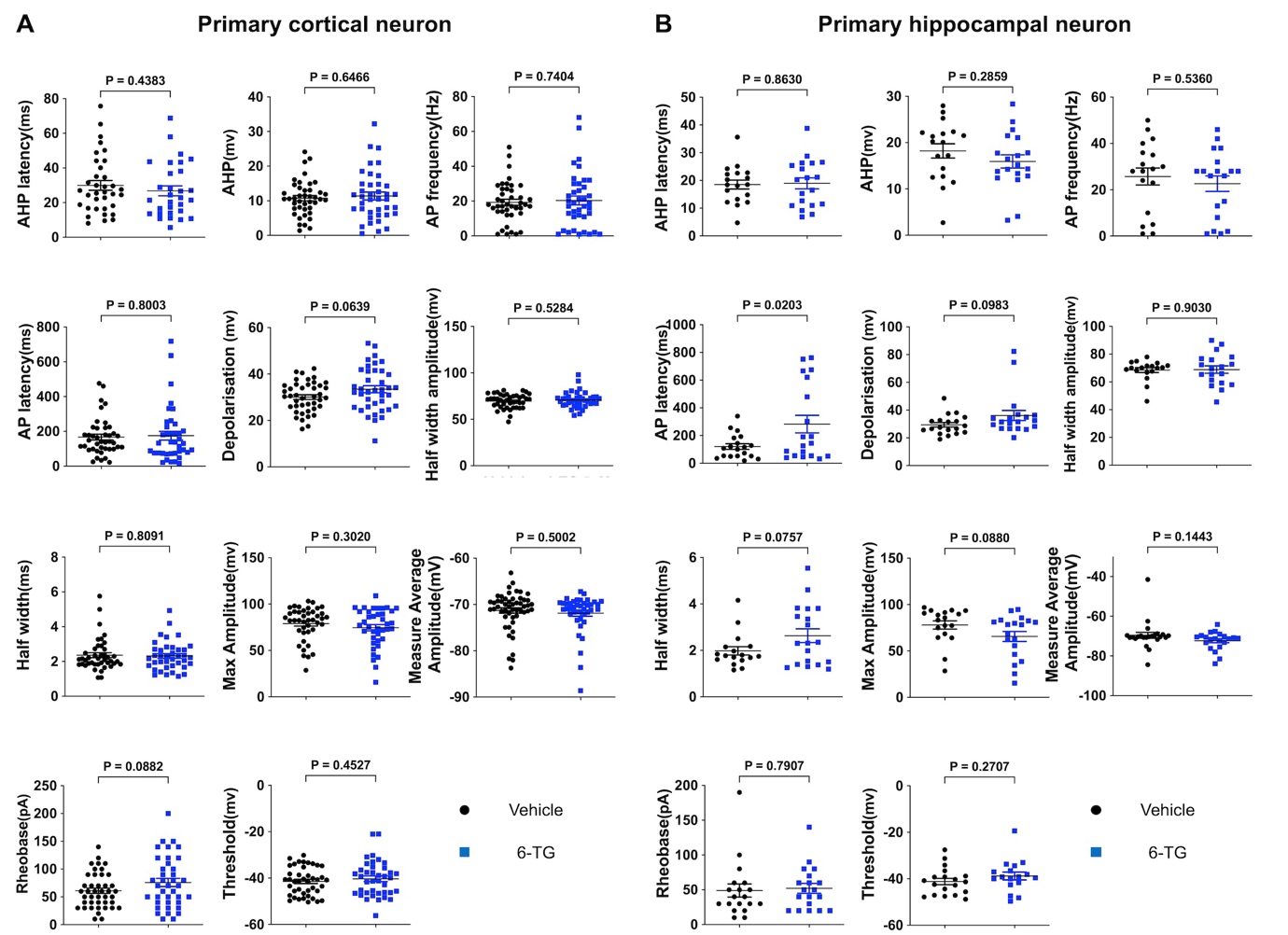


**Figure S3. 6-TG preserves the intrinsic properties of primary neuronal cells.** Mouse primary cortical and hippocampal neurons were exposed to 5 μM 6-TG for 24 h. **(A)** Analysis of primary cortical neurons. **(B)** Analysis of primary hippocampal neurons. Intrinsic properties were assessed and compared with raw data of each group, with values are presented as means (SD). Statistical significance was derived from unpaired t test relative to vehicle treated controls.


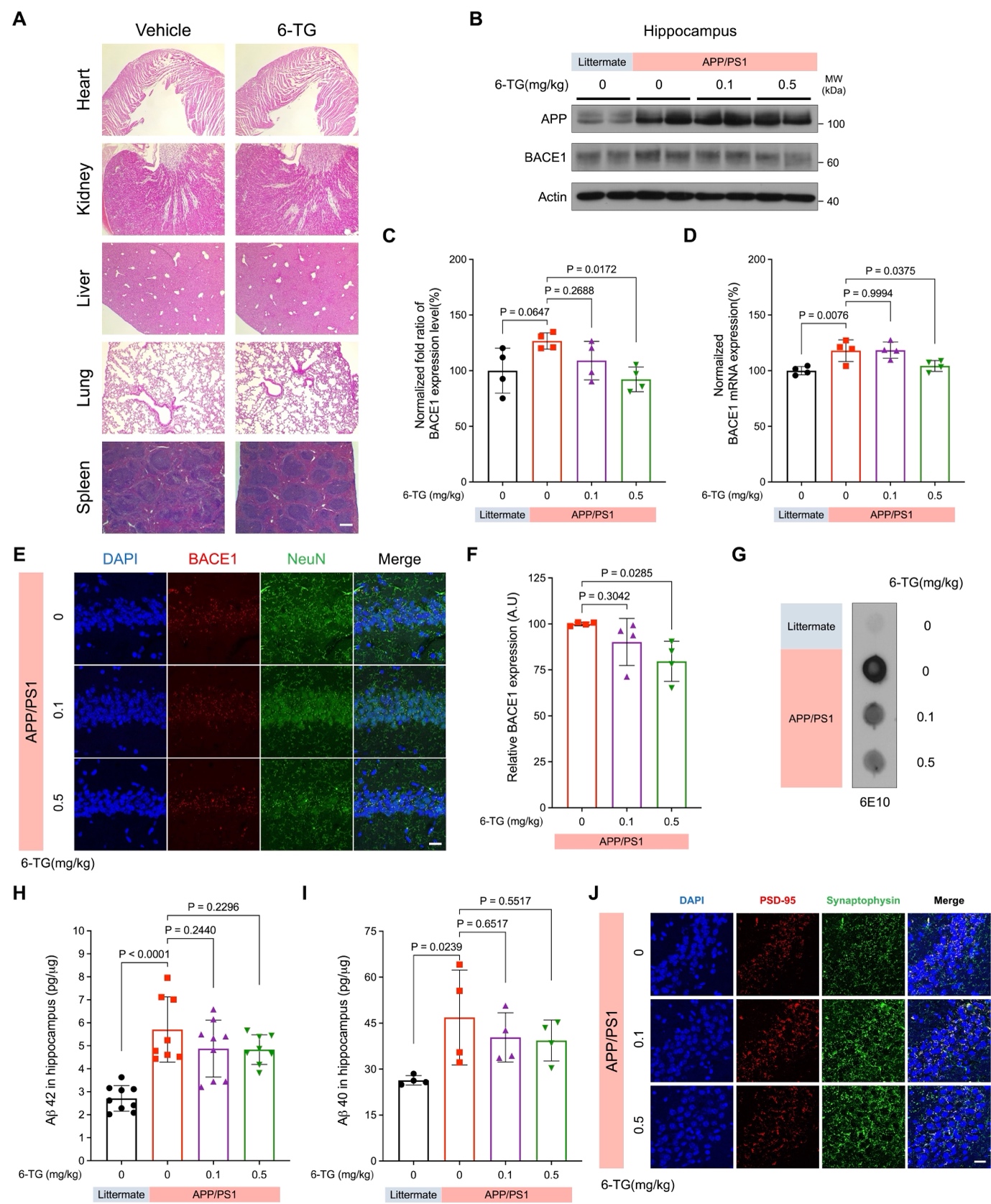


**Figure S4. Attenuation of AD pathology in the hippocampus of APP/PS1 mice by 6-TG. (A)** Histopathological analysis of major organs (heart, kidney, liver, lung, and spleen) following a 10-day oral administration of 6-TG at 2 mg/kg, a dose four times higher than the effective dose. No signs of toxicity, including apoptotic or necrotic events, were observed in any of the examined tissues. Scale bar = 400 μm **(B)** Protein expression in the hippocampus was assessed post-behavioral tests, with tissues homogenized in T-PER buffer and analyzed via immunoblotting for BACE1 and APP. **(C)** Quantification of BACE1 protein levels, normalized to β-actin. **(D)** BACE1 mRNA levels in hippocampal samples were quantified using RT-PCR, normalized to GAPDH, and compared across three primer sets to ensure accuracy relative to littermate controls. **(E)** Immunofluorescence imaging depicted BACE1 (red) and neuronal marker NeuN (green) in hippocampal sections (30μm) under a 40× magnification. Scale bar = 20 μm. **(F)** Quantitative analysis of BACE1 expression based on fluorescent imaging data. **(G)** Protein dot blot assay with 10μg of hippocampal extract probed with the 6E10 antibody to detect Aβ presence. **(H and I)** ELISA measured Aβ concentrations, establishing quantification against a standard curve. **(J)** Synaptic integrity in the CA3 hippocampal region of 6-TG-treated APP/PS1 mice, visualized via immunofluorescence. DAPI (blue) marks nuclei, PSD-95 (red) delineates postsynaptic density, and Synaptophysin (green) identifies presynaptic sites. Colocalization of PSD-95 and Synaptophysin indicates synaptic junctions. Scale bar = 20 μm. Data are presented as mean (SD). Statistical analysis was conducted using ordinary one-way ANOVA and Holm-Šídák's multiple comparisons test, with significance assessed against APP/PS1 vehicle-treated groups.


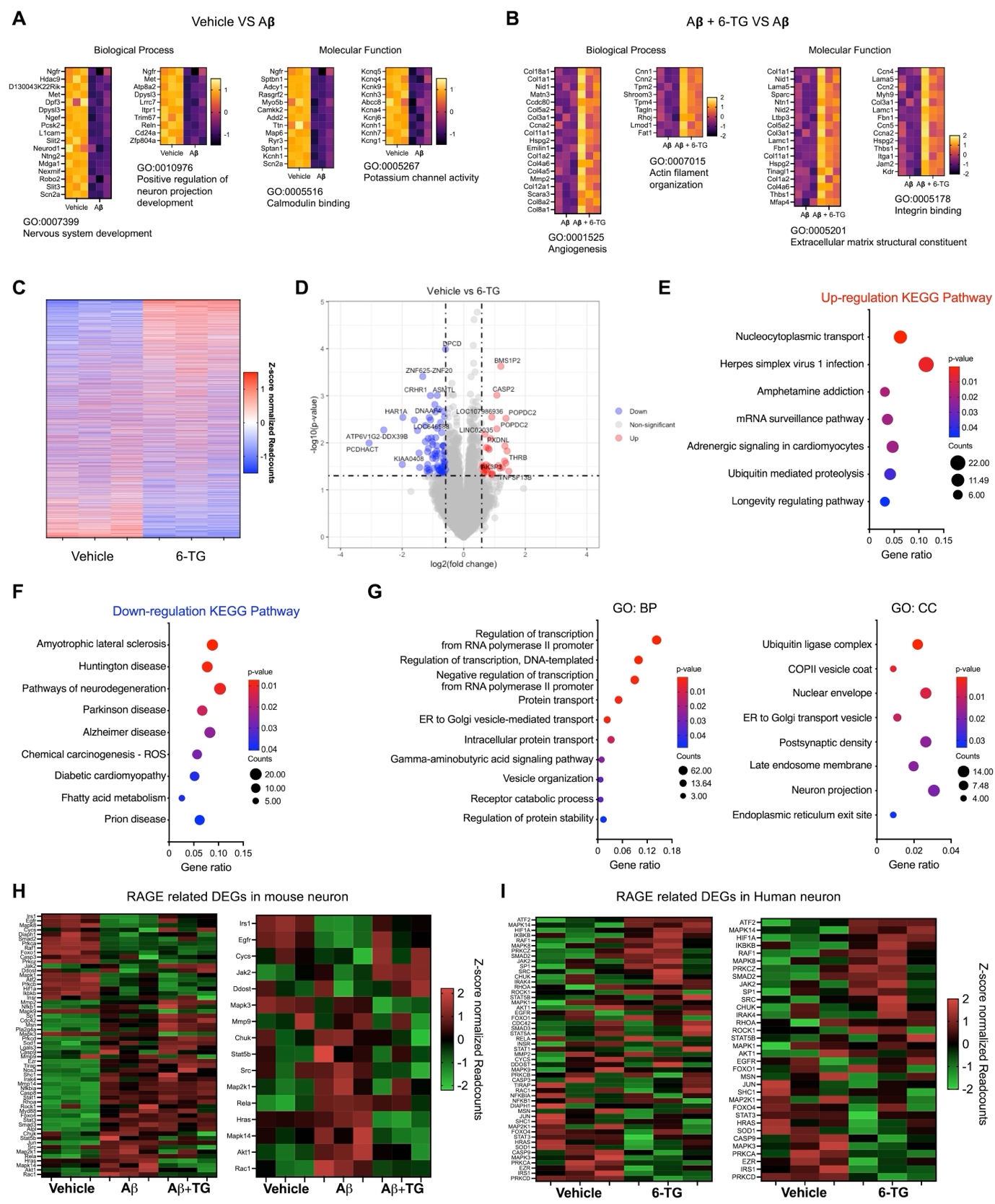


**Figure S5. Modulation of RAGE pathway-related gene expression by 6-TG in neuronal cells. (A and B)** GO analysis showing significantly altered biological processes and molecular functions affected by Aβ (A) or 6-TG treatment (B), represented by gene ratios and enrichment significance (-log FDR value). **(C)** Neuronal cells were treated with 5 μM 6-TG, followed by RNA extraction and sequencing. Heatmap depicting hierarchical clustering of DEGs among treatment groups. Expression levels are color-coded: red signifies upregulation, blue indicates downregulation, normalized to z-score readcounts. **(D)** Volcano plot demonstrates DEGs between vehicle- and 6-TG-treated neurons, with log2(fold change) and -log10(p-value) axis. Red points represent upregulated genes, blue points denote downregulated genes, and grey points signify non-significant changes. **(E and F)** KEGG pathway analysis showing significant pathways altered by 6-TG treatment in neurons, with pathway engagement quantified by P values and gene counts. **(G)** Gene Ontology (GO) analysis revealing top biological processes and cellular components affected, with gene ratios and enrichment significance (-log p-value) depicted. **(H)** In mouse primary neurons, a heatmap elucidates RAGE pathway-related gene expression patterns following treatment with vehicle, Aβ, or Aβ plus 6-TG. A gradient from green to red indicates expression levels from downregulated to upregulated genes. **(I)** In human embryonic stem cell-derived neurons post-NGN2 induction, a heatmap shows changes in RAGE pathway-associated genes after 6-TG treatment.


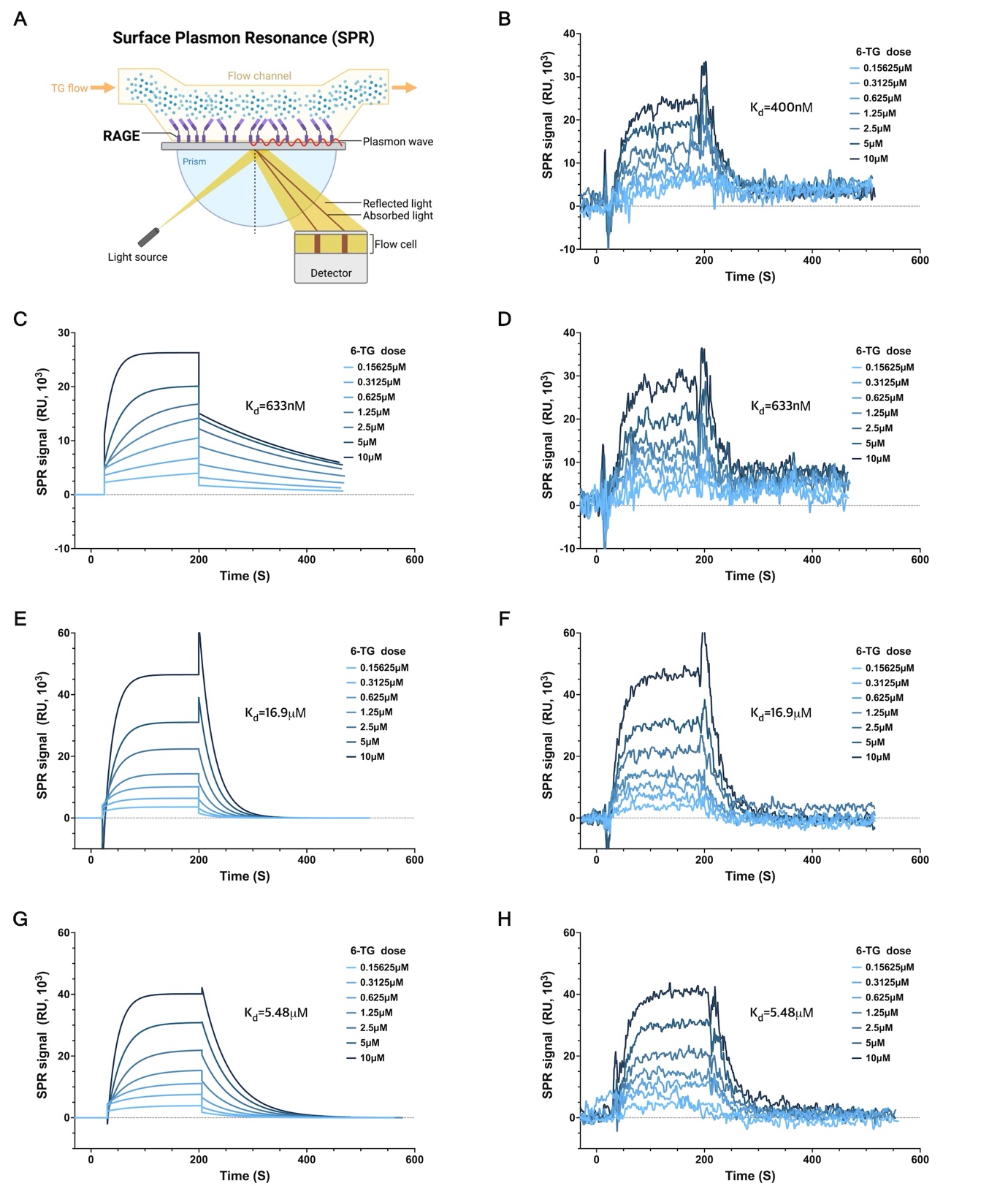


**Figure S6. SPR analysis of 6-TG interaction with recombinant RAGE proteins.** This figure illustrates the binding affinity and kinetics of 6-TG with various RAGE forms (Type1 RAGE: B to D, Type2 RAGE: E to H, The details of the RAGE recombinant protein are provided in the Methods section.) with Kd values reflecting interaction strength. **(A)** Schematic representation of the SPR assay, detailing the analysis of the interaction between 6-TG and RAGE proteins immobilized on the SPR sensor chip. **(B, D, F and H)** Display raw SPR data for 6-TG interactions with different RAGE variants, capturing the real-time binding dynamics, and showing the association and dissociation phases over various 6-TG concentrations. **(C, E and G)** Normalized SPR data presenting equilibrium binding analysis, depicting a direct correlation between 6-TG concentrations and response units (RU), facilitating a clear understanding of the binding interactions.


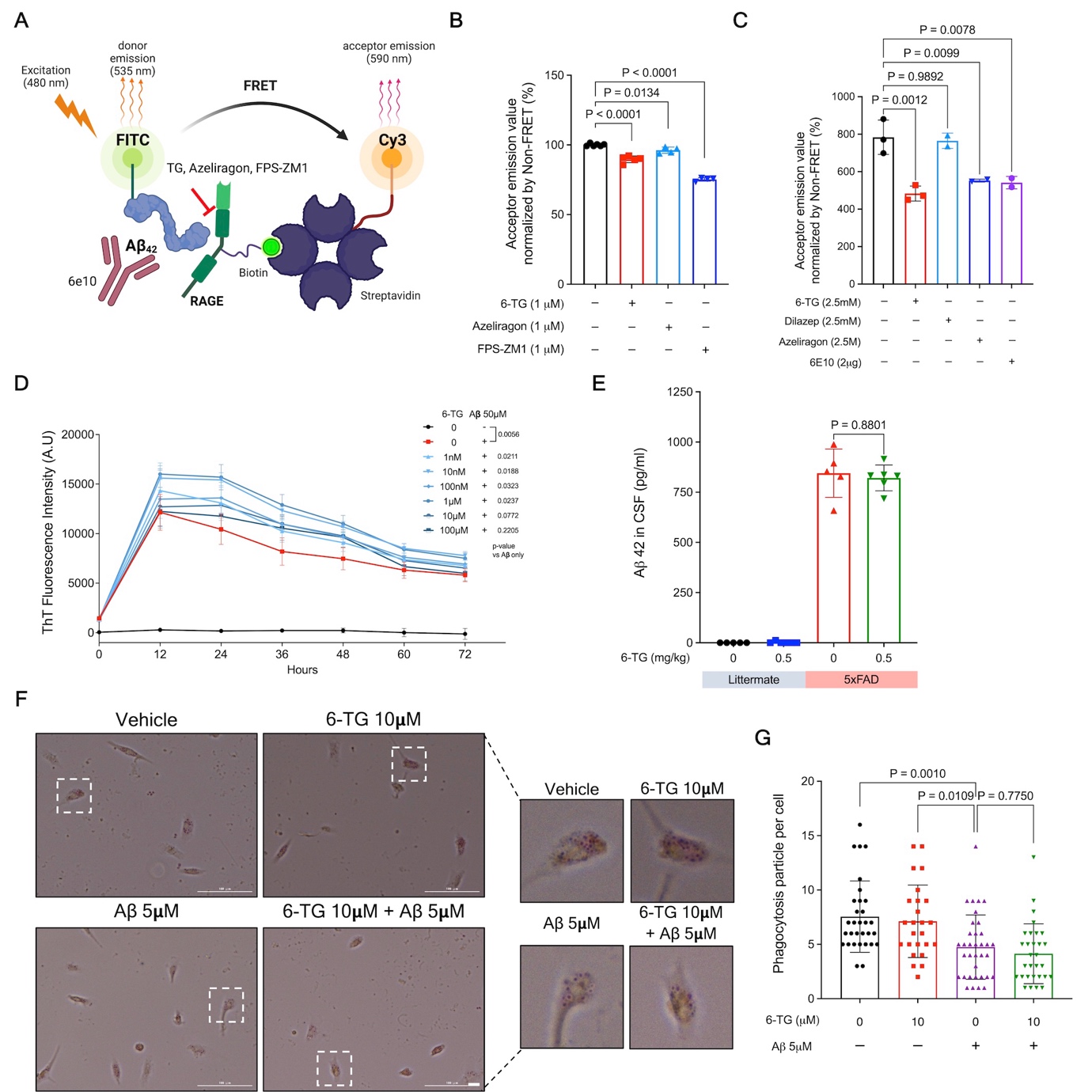


**Figure S7. Analyzing 6-TG interaction with RAGE and its impact on microglial phagocytosis. (A)** Schematic of the FRET assay setup, showing biotinylated RAGE protein tethered to Cy3-labeled streptavidin, and the intervention of 6-TG and RAGE antagonists (Azeliragon, FPS-ZM1) inhibiting the interaction between FITC-labeled Aβ and RAGE. Energy transfer upon RAGE-Aβ binding is monitored as a shift from FITC emission to Cy3 emission, excited at 480 nm. **(B and C)** Quantitative analysis of FRET efficiency, expressed as the ratio of Cy3 acceptor to FITC donor emission, reveals the interaction dynamics between 6-TG/Azeliragon/FPS-ZM1 and the RAGE-Aβ complex, with decreased FRET efficiency indicating effective 6-TG binding to RAGE. **(D)** ThT fluorescence assay results showing the effect of 6-TG on Aβ aggregation. Aβ (50 µM) was treated with varying concentrations of 6-TG. Fluorescence intensity was measured to evaluate the extent of Aβ aggregation in the presence of 6-TG. **(E)** ELISA quantification of Aβ_42_ level in CSF, employing standard curve comparison. Statistical analysis was conducted using ordinary one-way ANOVA and Holm-Šídák's multiple comparisons test, with significance assessed against 5xFAD vehicle-treated groups. **(F)** Representative micrographs of primary microglia ingesting pH-sensitive Zymosan BioParticles™, assessing the effect of 6-TG on Aβ-compromised phagocytic activity. Detailed imagery shows that 6-TG does not rectify the Aβ-induced phagocytic decline in RAGE KO microglia. Scale bar = 20 μm. **(G)** Phagocytic activity quantification across assays. Data are presented as mean (SD) with P values derived from ordinary one-way ANOVA and Dunnett’s multiple comparisons test, denoting statistical significance relative to vehicle treated controls (B and C). Ordinary one-way ANOVA and Holm-Šídák's multiple comparisons test, denoting statistical significance relative to Aβ only treated controls (E).


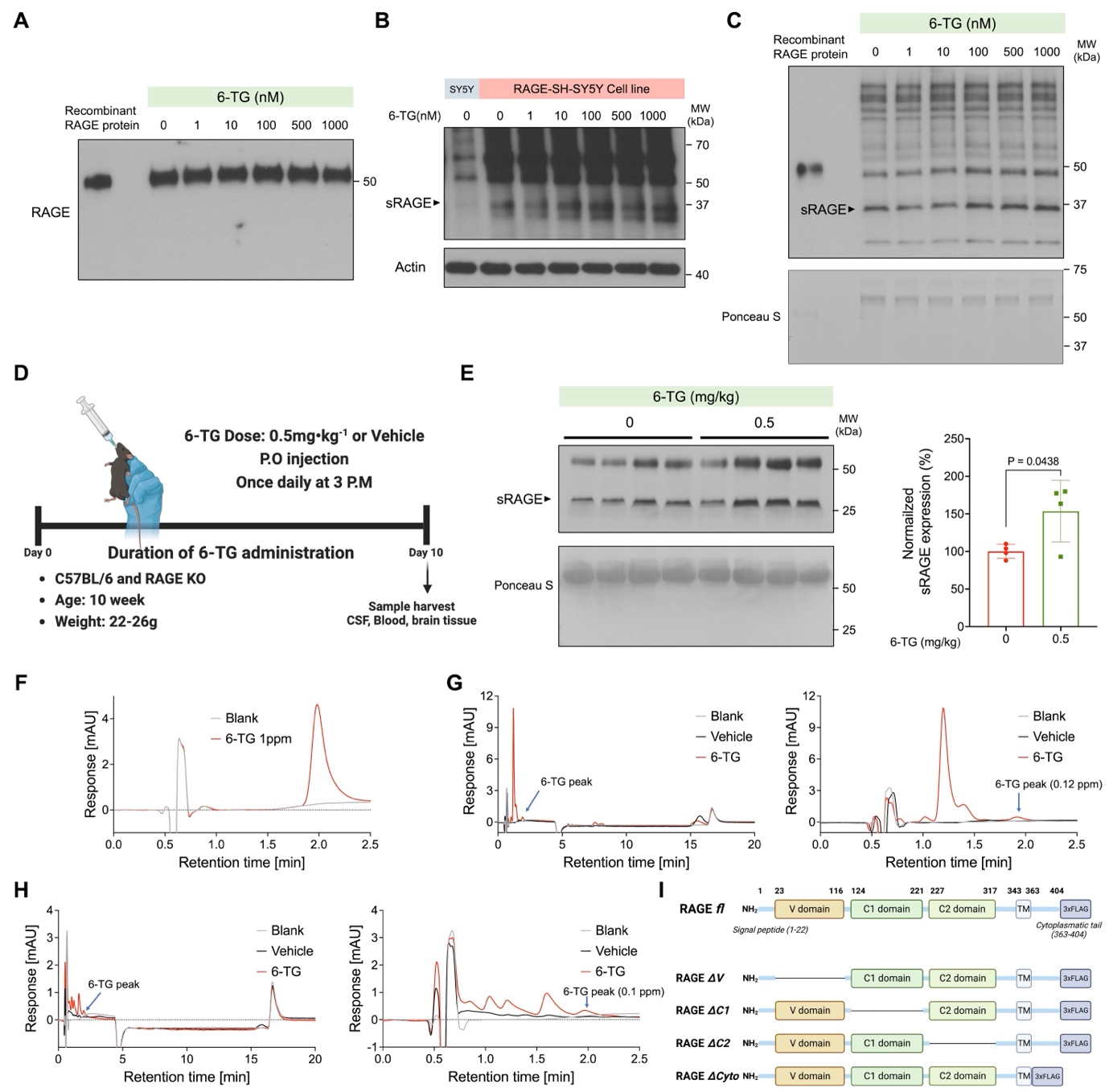


**Figure S8. sRAGE modulation by 6-TG in RAGE-overexpressing SH-SY5Y and *in vivo*. (A)** Evaluation of the effect of 6-TG on recombinant RAGE processing, testing its capacity to generate sRAGE, where no direct cleavage activity was detected under the experimental conditions. **(B)** Dose-response of 24 h 6-TG treatment on sRAGE production in RAGE-overexpressing SH-SY5Y, revealing cytosolic sRAGE accumulation at increasing concentrations of 6-TG. **(C)** Extracellular sRAGE quantification in the culture media of RAGE-overexpressing SH-SY5Y cells post 6-TG treatment for 24 h, indicating a dose-dependent enhancement in sRAGE secretion. **(D)** Overview of 6-TG administration in C57BL/6 mice, including dosage, treatment duration, and sampling timeline for CSF, blood, and brain tissues. **(E)** sRAGE levels in the plasma of C57BL/6 mice following 6-TG administration, with observed increases in sRAGE levels post-treatment. **(F)** Liquid chromatography (LC) analysis of 6-TG standard solution (1 ppm) in mobile phase solvent. A distinct peak for 6-TG is observed at approximately 2 minutes, establishing its retention time for subsequent identification in biological samples. **(G and H)** LC analysis confirming the presence of 6-TG in (G) plasma and (H) brain samples. The peak at approximately 2 minutes corresponds to 6-TG, indicating its detection in both samples following oral administration. **(I)** Schematic representation of RAGE variants, featuring full-length RAGE (RAGE-FL) and its truncated mutants lacking specific domains (∆V, ∆C1, ∆C2, ∆Cyto), each tagged with 3xFLAG for detection purposes.


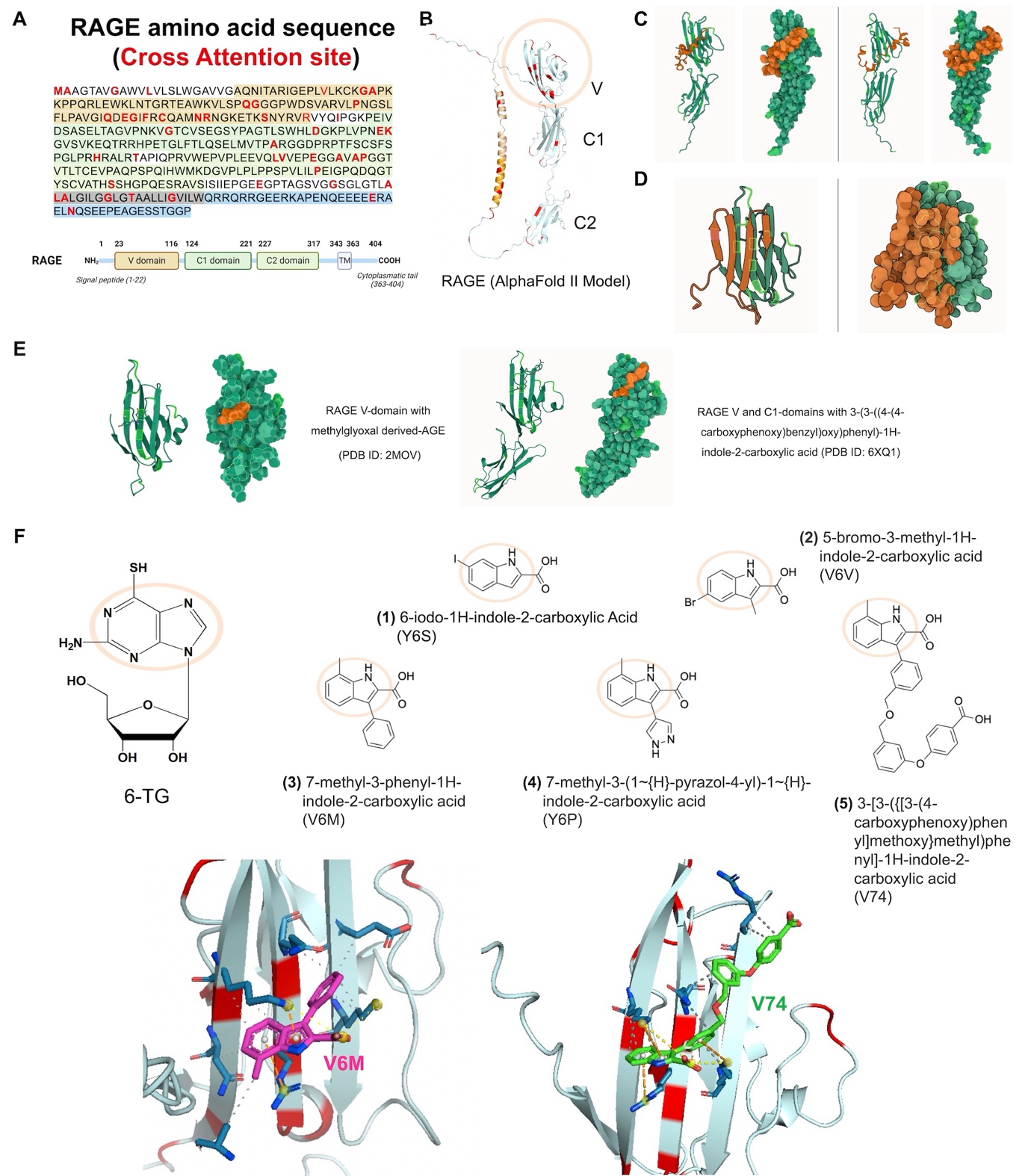


**Figure S9. Prediction of the binding site for RAGE-6-TG interaction. (A)** Amino acid sequence of RAGE with potential 6-TG interaction sites highlighted in red, particularly emphasizing the V domain where cross-attention data is concentrated, indicating key interaction redions. **(B)** Tertiary structure of RAGE, predicted by AlphaFold II (*76*), with regions cross-attention highlighted in red. This structure analysis aligns with the predicted ligand-binding sites, underscoring the interaction specificity within the V domain. **(C)** The RAGE-Aβ_42_ complex was predicted using ClusPro 2.0, based on experimentally validated structures of RAGE (V and C1 domains determined by X-ray crystallography; PDB ID: 3CJJ) and Aβ_42_ (determined by solution NMR; PDB42: 6SZF). **(D)** The RAGE (V domain) and Aβ_42_ complex was predicted using AlphaFold 3. The RAGE protein is shown in green, and the Aβ_42_ peptide is shown in red. Key residues in the RAGE protein, predicted to interact with 6-TG by the cross-attention mechanism of the MT-DTI deep learning model, are highlighted in bright green. **(E)** Structural analysis of RAGE V-domain interactions with methylglyoxal-derived AGEs and 3-(3-((4-(4-carboxyphenoxy)benzyl)oxy)phenyl)-1H-indole-2-carboxylic acid. The binding interactions were found to occur through similar residues as those predicted for 6-TG using the MT-DTI deep learning model. The overlap of interaction sites suggests a shared binding mechanism between 6-TG and other RAGE ligands, highlighting potential structural determinants for ligand recognition. **(F)** Structural comparison of various ligands, including 6-TG, that bind to the V domain of RAGE, showing similarities in purine structure. The diagram points out ligands with a common indole moiety conducive to pi-cation interactions within the V domain.


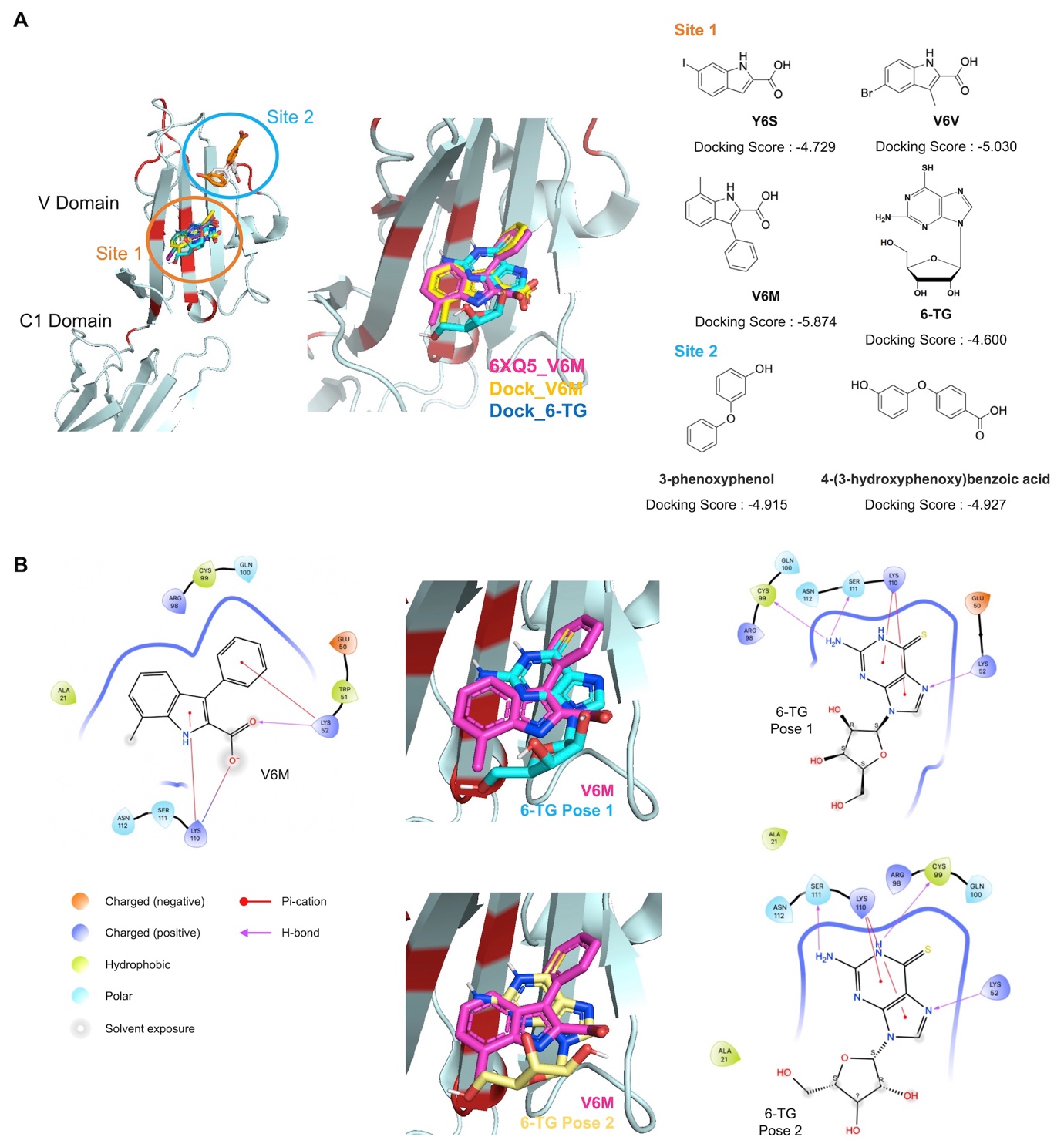


**Figure S10. Docking analysis of 6-TG interaction with RAGE. (A)** Docking simulations identify two potential 6-TG binding sites on RAGE, optimal docking scores obtained when the purine group of 6-TG aligns similarly to the indole group of the ligand. **(B)** Detailed docking poses reveal that the purine moiety of 6-TG is predicted to interact with RAGE, particularly through pi-cation interactions at residues K110 and K52. The left panel shows the interaction model of molecule V6M with RAGE, serving as a reference for the 6-TG-RAGE binding model. This analysis suggests that the most favorable binding occurs when the purine base of 6-TG coincides with the indole position of the ligand, engaging in significant pi-cation interactions with key amino acids (E50, K52, R98, Q100, K110, N112) at the RAGE V Domain binding site.

Table S1. Binding energies of small molecules with the RAGE V-domain

| **Small molecules** | **SMILES** | **Binding Energy (kcal/mol)** |
| --- | --- | --- |
| CHEMBL4072170 | c1ccc(Nc2ncc3nc[nH]c3n2)cc1 | -5.452 |
| CHEMBL1723172  (Different residues analogue) | C1C(C(OC1N2C=NC3=C2NC=NC3=S)CO)O | -5.4 |
| CHEMBL19940 | Nc1ncc2ncn(Cc3ccccc3)c2n1 | -5.386 |
| CHEMBL138342 | Nc1nc(SCc2ccccc2)c2[nH]cnc2n1 | -5.226 |
| CHEMBL448290 (Different residues analogue) | C1=NC(=S)C2=C(N1)N(C=N2)[C@H]3[C@@H]([C@@H]([C@H](O3)CO)O)O | -5.153 |
| CHEMBL3233216 | Cc1nc2c(=O)[nH]c(N)nc2[nH]1 | -5.093 |
| CHEMBL1504447  (Partially similar analogue) | CCCCN1C=NC2=C1NC(=NC2=S)N | -5.062 |
| CHEMBL608889 (6-TG) | C1=NC2=C(N1)C(=S)N=C(N2)N | -5.037 |
| CHEMBL1499973  (Partially similar analogue) | CC1(OC2C(OC(C2O1)N3C=NC4=C3NC=NC4=S)CO)C | -5.008 |
| CHEMBL305686  (Different residues analogue) | C1[C@H](O[C@H]([C@@H]1O)N2C=NC3=C(N=CN=C32)N)CO | -4.982 |
| CHEMBL1496167 | Nc1nc(S)c2ncn(C3CCCC3)c2n1 | -4.854 |
| CHEMBL1361836 | CCCn1cnc2c(S)nc(N)nc21 | -4.753 |
| CHEMBL3246422 | CNC(=S)Nc1ncnc2[nH]cnc12 | -4.615 |
| CHEMBL1999396 | CCn1cnc2c(=S)nc(N)[nH]c21 | -4.498 |
| CHEMBL4100771 | CN(C)c1nc(N)c2c(ncn2C)n1 | -4.259 |
| CHEMBL1200751  (Partially similar analogue) | C1=NC2=C(N1)C(=S)N=CN2.O | -3.97 |
| CHEMBL1495284 | N#CCCn1cnc2c(S)ncnc21 | -3.139 |

Table S2. qRT-PCR primer information

| **Primer Name** | **Sequence (5' to 3')** |
| --- | --- |
| human BACE1(77) | Forward GCC CAA GAA AGT GTT TGA AGC |
|  | Reverse GCC AGA AAC CAT CAG GGA ACT |
| human BACE1(78) | Forward AGG AGA AGG AGA GGG AGT A |
|  | Reverse CTG GTG AGT GGT TGT GTT |
| human BACE1(129) | Forward CTC CAG GGA GTC GTC AGG |
|  | Reverse TTC ATC AAC GGC TCC AAC |
| human β-actin | Forward AGG CCA ACC GCA AGA AG |
|  | Reverse ACA GCC TGG ATA GCA ACG ATC |
| mouse BACE1(34) | Forward CCC TTT CCT GCA TCG CTA C |
|  | Reverse TAC ACA CCC TTT CGG AGG TC |
| mouse BACE1(73) | Forward TCC TTC CGC ATC ACC ATC |
|  | Reverse ACA GTC GTC TTG GGA CGT G |
| mouse BACE1(104) | Forward TGC ATC GCT ACT ACC AGA GG |
|  | Reverse CAT GAG GGA TGC TCA CCA G |
| mouse β-actin | Forward GCT GTG CTA TGT TGC TCT A |
|  | Reverse CTC GTT GCC AAT AGT GAT GA |

Table S3. Antibody information

| **Antibody** | **Clone/Type** | **Reactivity** | **Source** | **Supplier** | **Cat No.** | **Dilution (Udage)** |
| --- | --- | --- | --- | --- | --- | --- |
| BACE1 | D10E5/ Monoclonal | Human, Mouse, Rat | Rabbit | Cell Signaling Techology | 5606 | 1:1000 (WB) 1:250 (IF) |
| APP  (𝚊-CTF, β-CTF) | C1/6.1/ Monoclonal | Human, Mouse, Rat | Mouse | Biolegend | 802801 | 1:1000 (WB) |
| RAGE (for human) | Polyclonal | Human, Mouse | Rabbit | abcam | ab30381 | 1:1000 (WB) |
| RAGE (for mouse) | Polyclonal | Mouse, Rat | Rabbit | Sigma-Aldrich | R5278 | 1:1000 (WB) |
| NLRP3 | D4D8T/ Monoclonal | Human, Mouse | Rabbit | Cell Signaling Techology | 15101 | 1:1000 (WB) |
| STAT3 | 79D7/ Monoclonal | Human, Mouse, Rat, Monkey | Rabbit | Cell Signaling Techology | 4904 | 1:1000 (WB) |
| pSTAT3 (Tyr705) | D3A7/ Monoclonal | Human, Mouse, Rat, Monkey | Rabbit | Cell Signaling Techology | 9145 | 1:1000 (WB) |
| STAT1 | Polyclonal | Human, Mouse, Rat, Monkey | Rabbit | Cell Signaling Techology | 9172 | 1:1000 (WB) |
| pSTAT1 (Tyr701) | 58D6/ Monoclonal | Human, Mouse | Rabbit | Cell Signaling Techology | 9167 | 1:1000 (WB) |
| JAK2 | D2E12/ Monoclonal | Human, Mouse, Rat | Rabbit | Cell Signaling Techology | 3230 | 1:1000 (WB) |
| pJAK2 (Tyr1007/1008) | Polyclonal | Human, Mouse, Rat | Rabbit | Merck Millipore | 07-606 | 1:1000 (WB) |
| FLAG | M2/ Monoclonal | All | Mouse | Sigma-Aldrich | F1804 | 1:1000 (WB) |
| PSD95 | D27E11/ Monoclonal | Human, Mouse, Rat | Rabbit | Cell Signaling Techology | 3450 | 1:250 (IF) |
| Synaptophysin | SY38/ Monoclonal | Human, Mouse, Rat | Mouse | abcam | ab8049 | 1:250 (IF) |
| Aβ | 6E10/ Monoclonal | Human | Mouse | Biolegend | 803004 | 1:1000 (WB)  1:250 (DAB) |
| NeuN | A60/ Monoclonal | Human, Mouse, Rat | Mouse | Merck Millipore | MAB377 | 1:300 (IF) |
| Actin | AC-15/ Monoclonal | Human, Mouse, Rat | Mouse | Sigma-Aldrich | A5441 | 1:5000 (WB) |
